# Scaling advantage over path-integral Monte Carlo in quantum simulation of geometrically frustrated magnets

**DOI:** 10.1038/s41467-021-20901-5

**Published:** 2021-02-18

**Authors:** Andrew D. King, Jack Raymond, Trevor Lanting, Sergei V. Isakov, Masoud Mohseni, Gabriel Poulin-Lamarre, Sara Ejtemaee, William Bernoudy, Isil Ozfidan, Anatoly Yu. Smirnov, Mauricio Reis, Fabio Altomare, Michael Babcock, Catia Baron, Andrew J. Berkley, Kelly Boothby, Paul I. Bunyk, Holly Christiani, Colin Enderud, Bram Evert, Richard Harris, Emile Hoskinson, Shuiyuan Huang, Kais Jooya, Ali Khodabandelou, Nicolas Ladizinsky, Ryan Li, P. Aaron Lott, Allison J. R. MacDonald, Danica Marsden, Gaelen Marsden, Teresa Medina, Reza Molavi, Richard Neufeld, Mana Norouzpour, Travis Oh, Igor Pavlov, Ilya Perminov, Thomas Prescott, Chris Rich, Yuki Sato, Benjamin Sheldan, George Sterling, Loren J. Swenson, Nicholas Tsai, Mark H. Volkmann, Jed D. Whittaker, Warren Wilkinson, Jason Yao, Hartmut Neven, Jeremy P. Hilton, Eric Ladizinsky, Mark W. Johnson, Mohammad H. Amin

**Affiliations:** 1 D-Wave Systems, Burnaby, BC Canada; 2 Google, Zurich, Switzerland; 3 Google, Venice, CA USA; 4 Department of Physics, Simon Fraser University, Burnaby, BC Canada

**Keywords:** Computational science, Phase transitions and critical phenomena, Quantum simulation

## Abstract

The promise of quantum computing lies in harnessing programmable quantum devices for practical applications such as efficient simulation of quantum materials and condensed matter systems. One important task is the simulation of geometrically frustrated magnets in which topological phenomena can emerge from competition between quantum and thermal fluctuations. Here we report on experimental observations of equilibration in such simulations, measured on up to 1440 qubits with microsecond resolution. By initializing the system in a state with topological obstruction, we observe quantum annealing (QA) equilibration timescales in excess of one microsecond. Measurements indicate a dynamical advantage in the quantum simulation compared with spatially local update dynamics of path-integral Monte Carlo (PIMC). The advantage increases with both system size and inverse temperature, exceeding a million-fold speedup over an efficient CPU implementation. PIMC is a leading classical method for such simulations, and a scaling advantage of this type was recently shown to be impossible in certain restricted settings. This is therefore an important piece of experimental evidence that PIMC does not simulate QA dynamics even for sign-problem-free Hamiltonians, and that near-term quantum devices can be used to accelerate computational tasks of practical relevance.

## Abstract (web summary)

Experimental demonstration of quantum speedup that scales with the system size is the goal of near-term quantum computing. Here, the authors demonstrate such scaling advantage for a D-Wave quantum annealer over analogous classical algorithms in simulations of frustrated quantum magnets.

## Introduction

Recent experiments on random circuit sampling in a superconducting quantum processor^1,2^ lend credence to the ultimate viability of quantum computing, but leave open the question of quantum advantage in practical applications. In absence of quantum error correction, the utility of gate-model quantum computers has yet to be determined^3^. Meanwhile, analog quantum simulations are an attractive proving ground for near-term quantum devices: the application can be chosen to suit the strengths of the platform, allowing the demonstration of a variety of engineered many-body quantum phenomena in noisy hardware. Examples include the Kibble-Zurek mechanism^4,5^, dynamical phase transitions^6,7^, many-body localization^8^, Coulomb blockade^9^, and magnetic phase transitions in quantum systems^10,11^. Having established the possibility of simulating complex quantum phenomena on a manufactured quantum device, as Feynman famously proposed^12^, one comes to the next question: can the quantum device confer a computational advantage?

We address this question using a superconducting flux-qubit quantum annealing (QA) processor^13^ to simulate frustrated quantum magnets. Statistics of these systems at thermal equilibrium can be estimated using quantum Monte Carlo (QMC) methods with no numerical sign problem^14^. Although there are many types of QMC, the most appropriate choice in this setting is path-integral Monte Carlo (PIMC)^15,16^—we discuss the limitations of other approaches in the Supplementary Information. Ever since the appearance of QA processors, PIMC has been promoted as a classical analog, and even as a simulator of QA^17–22^. PIMC was even shown to simulate QA dynamics for single-path incoherent tunneling through a barrier^23^, casting doubt on the ability of QA to offer a computational advantage over PIMC. Furthermore, no previous experiment to date has shown QA to systematically outperform PIMC-based simulations of QA in scaling^19,21^, although a scaling advantage has been shown to be possible in theory, for ideal QA^22,24,25^.

Here we provide experimental evidence that a QA processor can provide a computational advantage over PIMC in a quantum simulation task. As we tune parameters to make the simulation more difficult, the advantage of QA grows, showing a real-world example in which QA exploits dynamics not available to PIMC. Up to now, equilibration dynamics of QA in this simulation have been too fast to observe directly. However, in this experiment, we have found a slow unwinding mechanism relevant to equilibration and tied to important statistics of the models, which we can now measure within experimental timescales.

## Results

### Simulated quantum magnets

**Fig. 1:**
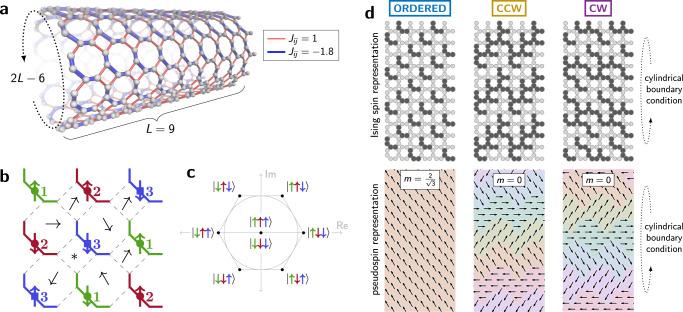
Geometrically frustrated lattice and escape from topological obstructions. **a** We program the superconducting processor to simulate a lattice with cylindrical boundary conditions, with lattice width *L* ∈ {6, 9, 12, 15}. Each square or octagonal plaquette is frustrated, having an odd number of antiferromagnetic (red) couplers that cannot be satisfied simultaneously. **b**, **c** Each plaquette is composed of spins in three sublattices (indicated by green, red, blue) (**b**). Due to frustration, each plaquette has six ground states, which map to six orientations of a pseudospin based on the value of spins in the three incident sublattices (**c**). Adding quantum fluctuations leads to ferromagnetic order in the pseudospins. **d** We initialize the simulation in three initial conditions: an ordered state, and states in which the pseudospin winds around the periodic dimension (top/bottom) counterclockwise (CCW) or clockwise (CW). Ordered states maximize the order parameter *m*, while CCW and CW have *m* = 0. Spin and pseudospin interpretations are shown. We simulate equilibration from these initial conditions using quantum (QA) and classical (PIMC) methods.

We simulate a square-octagonal lattice (Fig. 1a) in the transverse field Ising model (TFIM), whose Hamiltonian can be written as

1 $$H=H(s)=J(s)\sum _{i< j}{J}_{ij}{\sigma }_{i}^{z}{\sigma }_{j}^{z}-{{\Gamma }}(s)\sum _{i}{\sigma }_{i}^{x}$$

where *s* is a time-dependent annealing parameter, *J*_*ij*_ are 2-local coupling terms, *J*(*s*) is a global coupling energy prefactor, ${\sigma }_{i}^{z}$ and ${\sigma }_{i}^{x}$ represent Pauli operators on the *i*th spin, and Γ(*s*) is the transverse field, which induces quantum fluctuations. We study equilibration of the system at fixed *H*(*s*).

The lattice contains four-qubit ferromagnetic chains coupled together with antiferromagnetic bonds in a geometrically frustrated configuration^26,27^: no plaquette (square or octagon) can satisfy all its bonds simultaneously. Fully frustrated lattices are known to exhibit rich phase diagrams due to the interplay of quantum and thermal fluctuations^11,16,26^ (Supplementary Fig. 3). It was recently shown that the exotic physics of a topological phase transition in this system can be simulated in a QA processor^11^. However, the methods and apparatus did not allow accurate measurement of equilibration timescales. Here we introduce new experimental methods and apply them using an improved QA processor fabricated with lower-noise processes.

The lattice under study exhibits “order-by-disorder”, a phenomenon in which the introduction of the transverse field creates long-range order that does not exist in the classical setting^26^. Due to frustration in the lattice, each four- or eight-spin plaquette has six classical ground states; these can be represented by six orientations of a pseudospin (Fig. 1b, c). In the classical case (Γ = 0), these local ground states can combine to form a global ground state provided that neighboring pseudospins are approximately aligned, but this is not enough to impose long-range order^28^. If a four-qubit chain can be flipped without changing the energy, the addition of a small transverse field can put this chain in symmetric superposition of its “up” and “down” states: $(\left|\uparrow \uparrow \uparrow \uparrow \right\rangle +\left|\downarrow \downarrow \downarrow \downarrow \right\rangle )/\sqrt{2}$. Maximizing these energetically favorable configurations leads to long-range order, in which the pseudospins align ferromagnetically^11^ (Supplementary Discussion). Order is defined as the average pseudospin, captured by the complex order parameter

2 $$\psi =m\exp (i\theta )=({m}_{1}+{e}^{i2\pi /3}{m}_{2}+{e}^{i4\pi /3}{m}_{3})/\sqrt{3},$$

where *m*_1_, *m*_2_, and *m*_3_ are magnetizations of the three sublattices partitioning the lattice spins, indicated by color in Fig. 1b (also Supplementary Fig. 2). The phase diagram of the model has been established through analysis of the scaling of the real order parameter *m*, which varies as a function of transverse field Γ, temperature *T*, and lattice width *L* (ref. ^11^).

### Escape from topological obstruction

Our goal in this experiment is to measure the speed of equilibration in QA and PIMC simulations. A major limitation in probing QA dynamics is the lower bound on experimental timescales imposed by control circuitry^19,21^, in this case 1 μs. This limits our observations to slow equilibration processes. For the lattices under study, we have identified a slow mode in both QA and PIMC dynamics that can be attributed to topological winding in the pseudospin field. This slow equilibration signal can be amplified and observed clearly by initializing the simulation in a counterclockwise (CCW) or clockwise (CW) wound state, in addition to an ordered state with no topological obstruction (Fig. 1d and Supplementary Fig. 4). The pseudospin field rotates once (*m* = 0) or zero times ($m=2/\sqrt{3}$) around the periodic boundary direction in these initializations. The timescale associated with the unwinding/winding of these initial conditions—which requires global reconfiguration of the state—is captured in the convergence of *m* to its equilibrium distribution.

**Fig. 2:**
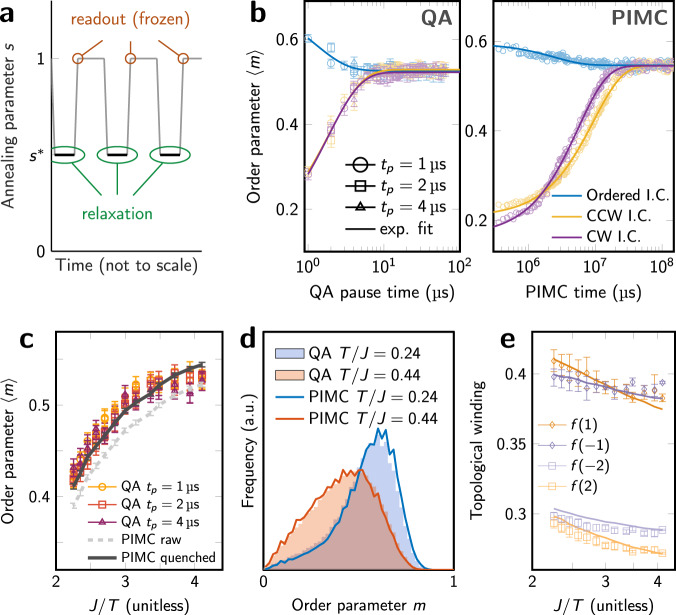
Convergence of statistical estimates for 1440-spin lattice. **a** The QA protocol alternates between equilibration and readout, forming a chain of annealing cycles, each containing a pause of length *t*_p_ during which relaxation occurs in the simulated model, parameterized by s^*^ (Supplementary Information). This breaks the simulation into discrete units, allowing observation of QA equilibration as in the Markov-chain PIMC simulation. **b** Starting from ordered, CCW, and CW initial conditions (Fig. 1), time-dependent QA and PIMC estimates of the order parameter $\left\langle m(t)\right\rangle$ converge to terminal values $\left\langle m\right\rangle$ for parameters Γ/*J* = 0.736 and *T*/*J* = 0.244. Fit lines show exponential convergence to equilibrium from each initial conditions. **c** QA estimates of $\left\langle m\right\rangle$ closely agree with quenched PIMC results (with local excitations removed) over a range of temperatures for Γ/*J* = 0.736, for two mappings of the lattice onto the QA processor. We attribute the underestimate of 0.01 at low temperatures to disorder in the Hamiltonian (Supplementary Information). **d** Histograms of *m* at high and low temperatures for Γ/*J* = 0.736 show accurate simulation of the entire distribution. **e** Topological winding from higher-order Fourier weights (Supplementary Information) shows that QA accurately simulates a subtle temperature-dependent preference between CCW (positive) and CW (negative) winding (lines = PIMC, symbols = QA). All error bars are 95% confidence interval on the mean.

In PIMC, convergence is quantized into Monte Carlo sweeps, providing a fine-grained time series of system evolution. In QA, we emulate this discrete process by using a “quantum evolution Monte Carlo” (QEMC) protocol^11^ that iterates over initialization, pause, and quench operations (Fig. 2a). We equilibrate Eq. (1) at annealing parameter s^*^ at device physical temperature *T* as follows: we initialize the QA processor in a specified classical state (Fig. 1d) at *s* = 1, where both quantum and thermal fluctuations are negligible and dynamics are frozen, and then rapidly reduce *s* to s^*^; we then pause, evolving according to the system parameters *H*(s^*^) and *T*, for a duration *t*_p_ ranging from 1 to 4 μs; we then quench the system, rapidly increasing *s* back to 1 and reading out a state projected to the computational basis. Iterating this process many times allows us to measure QA equilibration in steps, as a time series with microsecond resolution. Figure 2b shows this time-series convergence in terms of the average order parameter $\left\langle m(t)\right\rangle$ at time *t*, for QA and PIMC from each of the initial conditions to equilibrium.

The QA readout quench, a non-ideal approximation to projective readout, is a destructive process that eliminates entanglement and allows for some local relaxation of energy. However, as shown in the Supplementary Information (Supplementary Figs. 7 and 17), only the pause portion of the QEMC protocol contributes significantly to determining QA timescales, whereas the number and duration of initialize/quench interruptions has a small impact. Local relaxation in the quench boosts $\left\langle m\right\rangle$; to model this in PIMC, we apply a fast classical greedy descent to projected states, resulting in roughly a 0.02 increase in $\left\langle m\right\rangle$ that is recoverable (Supplementary Fig. 18). This simple model largely explains the discrepancy between QA and PIMC estimates (Fig. 2c). The remaining deviation between QA and PIMC we can attribute largely to disorder in the QA Hamiltonian, which suppresses $\left\langle m\right\rangle$ at low temperatures. As with initialization/quench, this weak disorder does not significantly affect dynamics on longer timescales (Supplementary Fig. 16). Up to these small corrections, QA faithfully simulates not only the mean value of *m*, but the entire distribution (Fig. 2d). Moreover, it accurately simulates subtle details of winding asymmetry as seen in the pseudospin Fourier transform (Fig. 2e). In the Supplementary Discussion, we explore accuracy of winding and two-point correlation decay in greater detail.

**Fig. 3:**
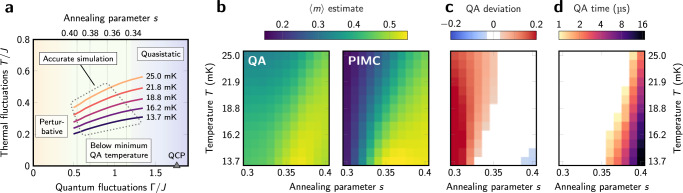
Region of accurate simulation and resolvable dynamics. **a** Our QA experiments are parameterized by annealing parameter *s* and physical temperature *T*; these map to the familiar (*T*/*J*, Γ/*J*) plane as shown. **b** Estimates of $\left\langle m\right\rangle$ are shown for QA and PIMC quenched samples for a range of *T* and *s*. **c** QA deviation from PIMC estimates is shown. When relaxation is extremely fast, order in the system is severely overestimated due to ordering during the readout quench. The white region falls within a tolerance of 0.03 of the ground truth, which corresponds to our “accurate simulation” region in **a**. At low *T* and high *s*, transverse field is small and ordering is weak, and therefore easily suppressed by inhomogeneities in the processor. **d** We cannot resolve convergence timescales faster than 1 μs. Our parameter range is therefore further restricted, excluding the fastest-converging models, i.e., those with high *T* and low *s*, shown in white.

We perform simulations over a range of QA parameters: annealing parameter s^*^ ranges from 0.30 to 0.40, and physical temperature *T* ranges from 13.7 mK to 25.0 mK. This maps to a region in the (Γ/*J*, *T*/*J*) plane (Fig. 3a). Figure 3b, c shows quenched QA and PIMC estimates of $\left\langle m\right\rangle$ and the deviation between them. Across a large swath of the parameter space, QA estimates of $\left\langle m\right\rangle$ agree with quenched PIMC estimates to within 0.03; we discard experiments outside this tolerance.

### Measuring computation time

The computational task performed by PIMC and QA is the equilibration of the simulated system with fixed Hamiltonian parameters. We quantify the speed of this process by measuring convergence of $\left\langle m(t)\right\rangle$ starting from initial states with CCW and CW winding (Fig. 1d)—this slow process is the computational bottleneck of the simulation. At the longest timescale, we find an exponential decay to provide a good fit (Fig. 2b), using the form

3 $$\left\langle m(t)\right\rangle =({m}_{0}-{m}_{f}){e}^{-t/\tau }+{m}_{f}$$

with fitting parameters *m*_0_, *m*_*f*_, and *τ*. To ensure quality of data, we measure convergence time *t* such that *m*(*t*) − *m*_*f*_ = 0.05. The use of a fit and threshold method allows a lower variance estimator of the timescale, but quantitatively similar results are found in using the exponent (*τ*) or a direct non-parametric estimate of time to threshold from the data (Supplementary Fig. 6). The geometric mean of CCW and CW convergence times, which are close, are presented in Fig. 3d. QA timescales less than the minimum experimental resolution of 1 μs are discarded. We emphasize that as in other experimental investigations of QA performance scaling, we have ignored times such as programming and readout which are not associated with the physical dynamics we attempt to quantify, since they give the false appearance of flat scaling.

**Fig. 4:**
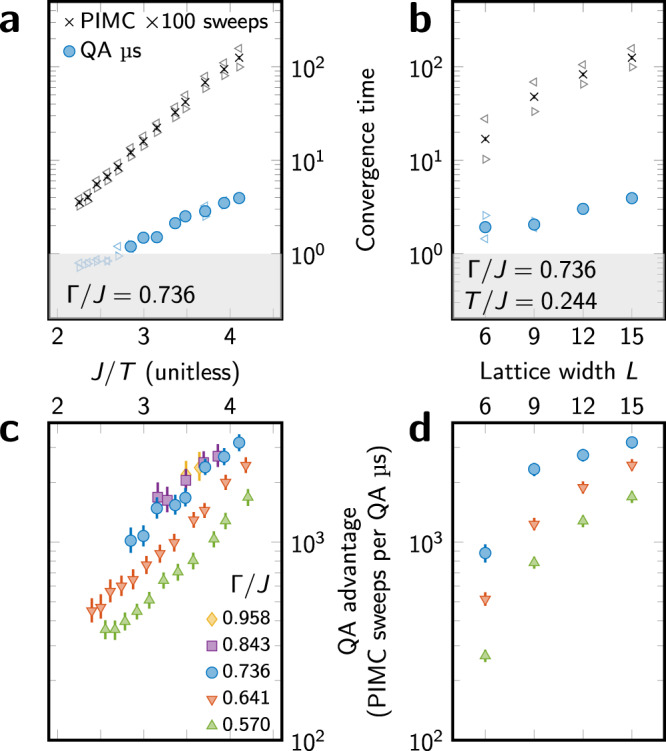
Scaling of convergence time and QA speedup. **a**, **b** Convergence time for both QA and PIMC as a function of inverse temperature *J*/*T* (**a**) and lattice width *L* (**b**). Triangles ⊲ and ⊳ indicate times for CCW and CW initial states, respectively; other markers indicate geometric mean. QA data are discarded if the estimate of $\left\langle m\right\rangle$ is not accurate to within 0.03, or either CCW or CW convergence time is <1 μs (shaded region). **c**, **d** QA advantage over PIMC, given as the ratio of convergence times, increases as *T* decreases (**c**), as quantum fluctuations increase (**c**), and as system size increases (**d**). Temperatures shown in **d** are minimum for which QA results are accurate (*J*/*T* ≈ 4.2 in each case). Scaling in *L* (**b**, **d**) is given in terms of PIMC sweeps to show equilibration dynamics rather than computation time. All filled data points have 95% CI bootstrap error bars, often smaller than the marker. At Γ/*J* = 0.736, *T*/*J* = 0.244, QA equilibration is three million times faster than PIMC on a CPU (Supplementary Information).

We now consider scaling of QA and PIMC convergence time as a function of temperature *T*, transverse field Γ, and lattice width *L* (Fig. 1a). We employ continuous-time PIMC code that updates four-qubit FM chains collectively, thereby obviating any local cluster bottleneck that might favor QA. This and potential alternatives are detailed in the Supplementary Discussion. Convergence timescales of both QA and PIMC increase as temperature decreases and as lattice width *L* increases (Fig. 4a, b). Testing the hypothesis that PIMC can act as a simulator of physical (QA) dynamics, we measure PIMC convergence in Monte Carlo sweeps, but find that QA and PIMC scaling differ significantly. The advantage of QA, given as the ratio of PIMC and QA convergence times, varies systematically not only in *T* and *L*, but also in Γ (Fig. 4c, d). Thus, the computational advantage conferred by the quantum hardware increases at harder parameterizations (increasing *L* and decreasing *T*) and as quantum mechanical effects increase (increasing Γ and decreasing *T*).

The scaling forms give us some information about quantum computational resources in this simulation. Both QA and PIMC scaling in *J*/*T* (Fig. 4a) resemble thermal activation *t* ∝ *e*^−*a*/*T*^ with different exponents *a*, indicating that equilibration is not dominated by a global tunneling event whose timescale saturates at low temperature. On the other hand, QA is clearly accessing resources beyond the four-qubit scale, otherwise the four-qubit updates in PIMC would negate any scaling advantage. Therefore, QA equilibration in this system is governed by quantum processes that are neither fully global nor fully local.

The slow escape from topological obstruction allows us to observe QA equilibration longer than 1 μs, which is crucial to the measurement of QA dynamics. We have examined alternative classical estimators and found PIMC to be the strongest classical competition among standard estimators for TFIMs (see Supplementary Information, Section 5).

## Discussion

We have experimentally demonstrated a computational scaling advantage over classical PIMC dynamics in simulating quantum magnetism with a programmable QA processor. In contrast to previous benchmarking studies^21,29,30^, our work shows not only a large absolute advantage, but also a scaling advantage for QA over the cluster-aware classical method in natural correspondence, on inputs that are of independent interest. Although PIMC is a leading classical approach to this type of simulation task, our study does not constitute a demonstration of superiority over all possible classical methods: there is a possibility that sophisticated algorithmic tricks or metaheuristic tools could accelerate PIMC. In the same vein, these tricks could be used to accelerate QA estimators in the form of hybrid algorithms. We expect the development and analysis of hybrid QA algorithms to be an active area of research in the coming years^3,31^.

These experiments provide evidence of a scaling advantage for QA over PIMC, and a detailed measurement of QA equilibration in a large frustrated system with high transverse field. This is essential evidence supporting the viability of QA as a computing platform. Far from being an artificial benchmark, the simulated lattice demonstrates the exotic topological phenomena that can arise in frustrated quantum Ising systems^11^. As Monte Carlo inference is an effective tool in the study of both idealized frustrated systems^16,26^ and real frustrated magnetic compounds such as Ca_3_Co_2_O_6_ (ref. ^32^) and TmMgGaO_4_ (ref. ^33^), our experiment is closely related to real-world applications.

These results constitute an encouraging milestone: a programmable quantum system can simulate quantum condensed matter far faster than the corresponding classical method, and with better scaling in problem size and hardness. Extensions of this work abound: related phenomena of great interest include material properties in the vicinity of a quantum critical point^34^, and dynamics of monopole excitations in artificial quantum spin ice^35,36^. Simulating these near the zero-temperature limit in QA would benefit from processors with more flexible lattice connectivity, higher coupling energy, lower noise, and improved projective readout. These programmable quantum simulations may ultimately be applied to the design of exotic new materials that are just beyond the computational horizon. The advantage reported in this work also opens the door to hybrid approaches that could be used to accelerate high-performance computing tasks. As various quantum computing technologies mature, we anticipate similar scaling advantages in the simulation of quantum systems—such demonstrations are crucial waypoints for the field as a whole.

## Supplementary information

Supplementary Information

## Data Availability

The datasets generated and analyzed during the current study are available from the corresponding author on reasonable request.

## Code availability

Simplified PIMC code is available at https://github.com/dwavesystems/dwave-pimc. The open-source implementation is discussed in the Supplementary Information.

## References

[1] Arute F (2019). Quantum supremacy using a programmable superconducting processor. Nature.

[2] Pednault, E., Gunnels, J. A., Nannicini, G., Horesh, L. & Wisnieff, R. Leveraging secondary storage to simulate deep 54-qubit sycamore circuits. Preprint at http://arxiv.org/abs/1910.09534 (2019).

[3] Preskill J (2018). Quantum computing in the NISQ era and beyond. Quantum.

[4] Keesling A (2019). Quantum Kibble-Zurek mechanism and critical dynamics on a programmable Rydberg simulator. Nature.

[5] Bando Y (2020). Probing the universality of topological defect formation in a quantum annealer: Kibble-Zurek mechanism and beyond. Phys. Rev. Res..

[6] Zhang J (2017). Observation of a many-body dynamical phase transition with a 53-qubit quantum simulator. Nature.

[7] Jurcevic P (2017). Direct observation of dynamical quantum phase transitions in an interacting many-body system. Phys. Rev. Lett..

[8] Roushan P (2017). Spectroscopic signatures of localization with interacting photons in superconducting qubits. Science.

[9] Hensgens T (2017). Quantum simulation of a Fermi-Hubbard model using a semiconductor quantum dot array. Nature.

[10] Harris R (2018). Phase transitions in a programmable spin glass simulator. Science.

[11] King AD (2018). Observation of topological phenomena in a programmable lattice of 1,800 qubits. Nature.

[12] Feynman RP (1982). Simulating physics with computers. Int. J. Theor. Phys..

[13] Johnson MW (2011). Quantum annealing with manufactured spins. Nature.

[14] Loh EY (1990). Sign problem in the numerical simulation of many-electron systems. Phys. Rev. B.

[15] Suzuki M (1976). Relationship between d-dimensional quantal spin systems and (d+1)-dimensional Ising systems: equivalence, critical exponents and systematic approximants of the partition function and spin correlations. Prog. Theor. Phys..

[16] Isakov SV, Moessner R (2003). Interplay of quantum and thermal fluctuations in a frustrated magnet. Phys. Rev. B.

[17] Kadowaki T, Nishimori H (1998). Quantum annealing in the transverse Ising model. Phys. Rev. E.

[18] Boixo S (2014). Evidence for quantum annealing with more than one hundred qubits. Nat. Phys..

[19] Rønnow TF (2014). Defining and detecting quantum speedup. Science.

[20] Brady LT, van Dam W (2016). Quantum Monte Carlo simulations of tunneling in quantum adiabatic optimization. Phys. Rev. A.

[21] Albash T, Lidar DA (2018). Demonstration of a scaling advantage for a quantum annealer over simulated annealing. Phys. Rev. X.

[22] Mbeng GB, Privitera L, Arceci L, Santoro GE (2019). Dynamics of simulated quantum annealing in random Ising chains. Phys. Rev. B.

[23] Isakov SV (2016). Understanding quantum tunneling through quantum Monte Carlo simulations. Phys. Rev. Lett..

[24] Andriyash, E. & Amin, M. H. Can quantum Monte Carlo simulate quantum annealing? Preprint at https://arxiv.org/abs/1703.09277 (2017).

[25] Kechedzhi, K. et al. Efficient population transfer via non-ergodic extended states in quantum spin glass. In *13th Conference on the Theory of Quantum Computation, Communication and Cryptography (TQC 2018)*, *Leibniz International Proceedings in Informatics (LIPIcs)*, Vol. 111 (ed. Jeffery, S.) 9:1–9:16 (Schloss Dagstuhl-Leibniz-Zentrum fuer Informatik, Dagstuhl, Germany, 2018).

[26] Moessner R, Sondhi SL (2001). Sondhi, Ising models of quantum frustration. Phys. Rev. B.

[27] Moessner R, Ramirez AP (2006). Geometrical frustration. Phys. Today.

[28] Wannier GH (1950). The triangular Ising net. Phys. Rev..

[29] Denchev VS (2016). What is the computational value of finite-range tunneling?. Phys. Rev. X.

[30] Mandrà S, Zhu Z, Wang W, Perdomo-Ortiz A, Katzgraber HG (2016). Strengths and weaknesses of weak-strong cluster problems: a detailed overview of state-of-the-art classical heuristics versus quantum approaches. Phys. Rev. A.

[31] Morley JG, Chancellor N, Bose S, Kendon V (2019). Quantum search with hybrid adiabatic-quantum-walk algorithms and realistic noise. Phys. Rev. A.

[32] Kamiya Y, Batista CD (2012). Formation of magnetic microphases in Ca_3_Co_2_O_6_. Phys. Rev. Lett..

[33] Li H (2020). Kosterlitz-Thouless melting of magnetic order in the triangular quantum Ising material TmMgGaO_4_. Nat. Commun..

[34] Sachdev, S. *Quantum Phase Transitions* (Cambridge University Press, 2011).

[35] Henry L-P, Roscilde T (2014). Order-by-disorder and quantum Coulomb phase in quantum square ice. Phys. Rev. Lett..

[36] Farhan A (2019). Emergent magnetic monopole dynamics in macroscopically degenerate artificial spin ice. Sci. Adv..

