# Supplementary material for: Scaling advantage over path-integral Monte Carlo in quantum simulation of geometrically frustrated magnets
Source: Nat Commun. 2021 Feb 18;12:1113. doi: 10.1038/s41467-021-20901-5 (PMC7892843; doi:10.1038/s41467-021-20901-5)
Supplement: Supplementary file 1 — Supplementary Information [file 41467_2021_20901_MOESM1_ESM.pdf]

Supplementary Information
Scaling advantage over path-integral Monte Carlo in quantum simulation of geometrically frustrated magnets

Andrew D. King,[1,*] Jack Raymond,[1,†] Trevor Lanting,[1,†] Sergei V. Isakov,[2] Masoud Mohseni,[3] Gabriel Poulin-Lamarre,[1] Sara Ejtemaee,[1] William Bernoudy,[1] Isil Ozfidan,[1] Anatoly Yu. Smirnov,[1] Mauricio Reis,[1] Fabio Altomare,[1] Michael Babcock,[1] Catia Baron,[1] Andrew J. Berkley,[1] Kelly Boothby,[1] Paul I. Bunyk,[1] Holly Christiani,[1] Colin Enderud,[1] Bram Evert,[1] Richard Harris,[1] Emile Hoskinson,[1] Shuiyuan Huang,[1] Kais Jooya,[1] Ali Khodabandelou,[1] Nicolas Ladizinsky,[1] Ryan Li,[1] P. Aaron Lott,[1] Allison J. R. MacDonald,[1] Danica Marsden,[1] Gaelen Marsden,[1] Teresa Medina,[1] Reza Molavi,[1] Richard Neufeld,[1] Mana Norouzpour,[1] Travis Oh,[1] Igor Pavlov,[1] Ilya Perminov,[1] Thomas Prescott,[1] Chris Rich,[1] Yuki Sato,[1] Benjamin Sheldan,[1] George Sterling,[1] Loren J. Swenson,[1] Nicholas Tsai,[1] Mark H. Volkmann,[1] Jed D. Whittaker,[1] Warren Wilkinson,[1] Jason Yao,[1] Hartmut Neven,[3] Jeremy P. Hilton,[1] Eric Ladizinsky,[1] Mark W. Johnson,[1] and Mohammad H. Amin[1,4]

[1] *D-Wave Systems, Burnaby, British Columbia, Canada*
[2] *Google, 8002 Zurich, Switzerland*
[3] *Google, Venice, California 90291, USA*
[4] *Department of Physics, Simon Fraser University*

(Dated: December 17, 2020)

CONTENTS

1. Geometrically-frustrated Ising model and initial conditions 2
   a. Triangular antiferromagnet and order-by-disorder 2
   b. Square-octagonal lattice 4
   c. Initial conditions for the simulation 5
   d. Winding number 5
2. Quantum annealing processor 6
   a. Extracted QA timescales 8
   b. Insensitivity to quench rate 8
3. Measurement of QA parameters 8
   a. Qubit model parameters 10
   b. Low Noise Processor 10
   c. Qubit temperature 11
   d. Background susceptibility and compensation 11
   e. Effective spin-1/2 Hamiltonian 11
4. Path-integral Monte Carlo methods 12
   a. Discrete-time PIMC and Trotter error 13
   b. PIMC timing 14
   c. Wall-clock time and power usage 14
5. Competing methods: alternatives to PIMC 16
   a. Alternative MCMC methods: Cluster algorithms and stochastic series expansion 17
6. Competing methods: simulation via approximate models 19
   a. Six-state clock and other phenomenological approximations 19
   b. Triangular lattice transverse field Ising model approximation 20
   c. Spin vector model 20
7. Effect of quench and disorder 21
   a. Effect of quench on relaxation timescales 25
8. Local signatures of entanglement 25
9. Additional observables 27
10. Source code 27

1. Geometrically-frustrated Ising model and initial conditions

The magnetic system studied herein is a square-octagonal lattice whose spins are partitioned into four-spin ferromagnetically (FM) coupled chains (Fig. 1a). The chains are coupled to one another antiferromagnetically (AFM). Ferromagnetic and antiferromagnetic couplings have respective strengths of $J_{ij} = -1.8$ and $J_{ij} = 1$. Each plaquette of the lattice (square or octagon) has three antiferromagnetic bonds, meaning that in any classical spin state, each plaquette has at least one frustrated bond. One very desirable property of the square-octagonal lattice is that it can be embedded directly (without a multi-qubit approximation to single qubits) into the "Chimera"[1] qubit connectivity of the QA processor (see Supplementary Fig. 5).

In the classical ground state of the square-octagonal lattice the ferromagnetic chain bonds are never frustrated. By contracting each four-qubit chain down into a single spin (Supplementary Fig. 3a–c) we can transform the square-octagonal lattice into the triangular antiferromagnet. Therefore to describe the statistical mechanics of the square-octagonal lattice we begin with the triangular antiferromagnet, which has the same low-energy description.

a. Triangular antiferromagnet and order-by-disorder

The triangular antiferromagnet is an archetypal example of geometric frustration, and has been widely studied both in the quantum case[3–8] and earlier in the related stacked magnet[9–11]. In the classical case (i.e., with $\Gamma = 0$), it does not exhibit long-range order but is critical at $T = 0$ [12].

To understand the ordering influence of the transverse field we begin with a single AFM triangle. In the classical Ising model this system has six ground states: $\uparrow\uparrow\downarrow$, $\uparrow\downarrow\uparrow$, $\downarrow\uparrow\uparrow$, $\uparrow\downarrow\downarrow$, $\downarrow\uparrow\downarrow$, $\downarrow\downarrow\uparrow$ (Supplementary Fig. 2a). Adding a small transverse field Γ results in six different ground states (to first order perturbation in Γ): $|\uparrow\uparrow\updownarrow\rangle$, $|\uparrow\downarrow\updownarrow\rangle$, $|\downarrow\updownarrow\uparrow\rangle$, $|\uparrow\updownarrow\downarrow\rangle$, $|\updownarrow\uparrow\downarrow\rangle$, $|\updownarrow\downarrow\uparrow\rangle$, where $\updownarrow$ indicates a spin in symmetric superposition of up and down: $(|\uparrow\rangle + |\downarrow\rangle)/\sqrt{2}$, aligned with the transverse field and having $\sigma^z = 0$ and $\sigma^x = 1$. In addition to the energetic contribution of the classical Ising portion of the Hamiltonian, the transverse field contributes $-\Gamma$ to the ground state energy due to the transverse-field-aligned spin. Extending these states from individual triangular plaquettes to the triangular lattice, we can tile any one of these six perturbative ground states across the lattice such that each triangle has one spin up, one spin down, and one spin aligned with the transverse field, giving a ground state of the entire triangular lattice (Supplementary Fig. 2d).

Excitations in the lattice can be understood through a mapping from an Ising spin state to a state of complex "pseudospin" rotors on the lattice plaquettes (Supplementary Fig. 2b–c). Given a perturbative ground state of the lattice as described above (Supplementary Fig. 2d), we note that the spins of the lattice are partitioned into three sublattices based on spin value: one up, one down, and one transverse field aligned. This allows us to map a spin state to a set of complex rotors on each plaquette $\mathcal{P}_j$ as follows. Denoting the three sublattices as S_1, S_2, S_3 and the Pauli z-operators on the spins of a triangle in each of these sublattices as σ^z_{j1}, σ^z_{j2}, σ^z_{j3} respectively, we define the plaquette's pseudospin as

$$\psi_j = \left(\sigma^z_{j1} + e^{2\pi i/3}\sigma^z_{j2} + e^{4\pi i/3}\sigma^z_{j3}\right)/\sqrt{3}, \tag{1}$$

where σ^z gives magnetization in the z-basis (Supplementary Fig. 2c). We can map both classical and quantum spin states of a plaquette onto complex rotors as shown in Supplementary Fig. 2b–c. The three-sublattice long range order for a lattice can be quantified by the average of ψ_j over all plaquettes; within order $1/L$ corrections from the

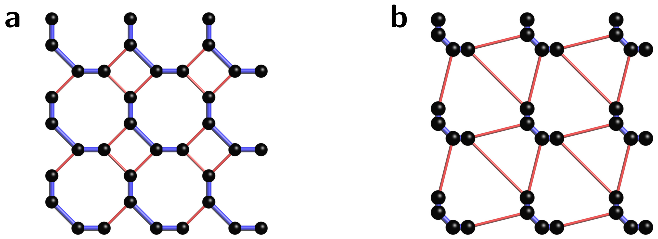

Supplementary FIG. 1. **Square-octagonal and triangular geometrically-frustrated lattices.** In this work we simulate a square-octagonal lattice (**a**) containing four-spin ferromagnetic chains (blue) coupled together antiferromagnetically (red). When each chain is contracted (**b**) into a single spin (**c**), the result is the widely-studied triangular antiferromagnet. The low-energy physics and phase diagram of the square-octagonal transverse field Ising model can be understood by analogy to the triangular case, as established in previous work[2].

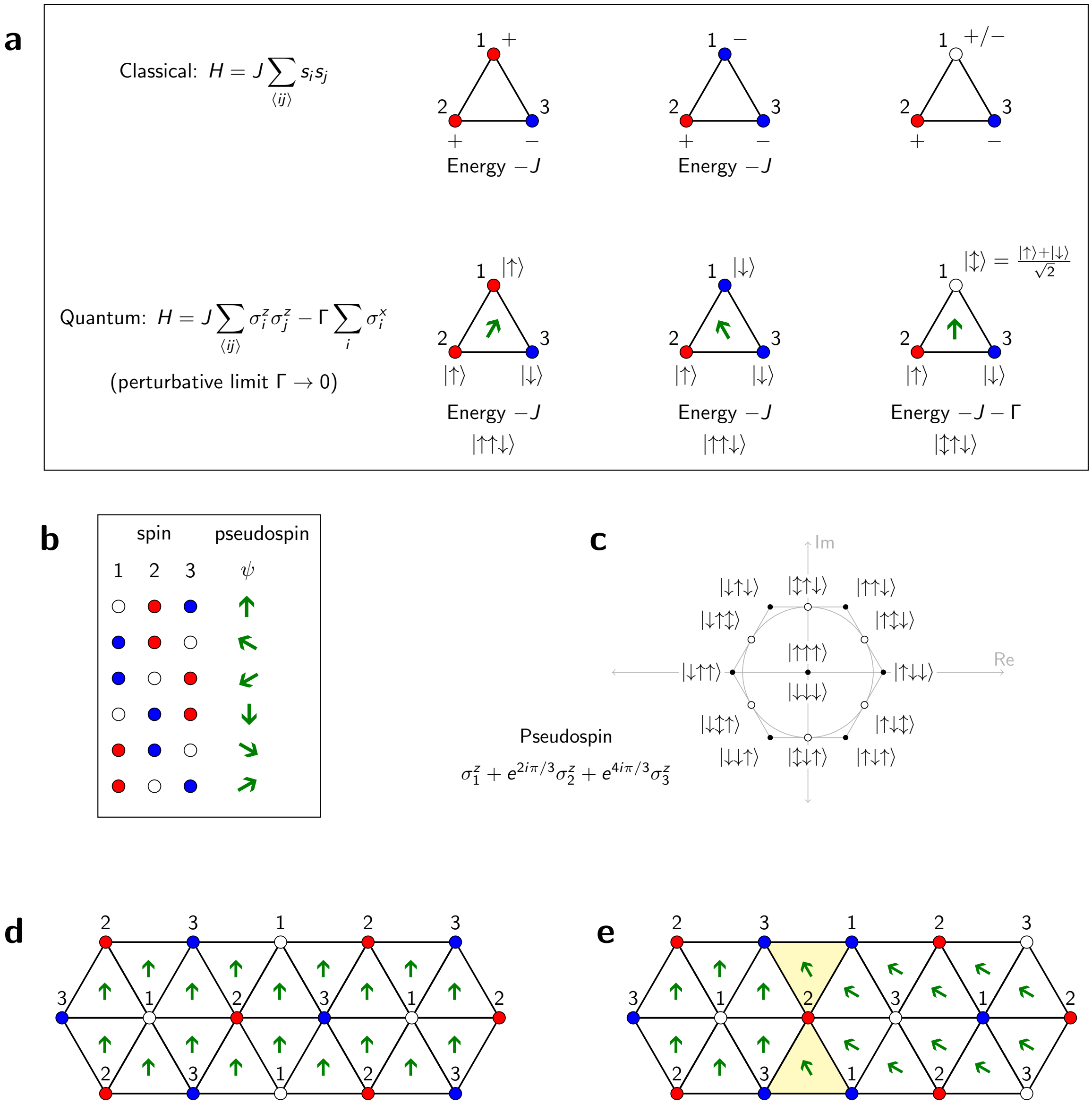

Supplementary FIG. 2. **Ordering effect of transverse field.** **a**, Each triangular plaquette has six degenerate ground states in the classical case. Two such ground states can differ by a single spin flip, resulting in a "flippable" spin that does not affect the energy. A small transverse field compels a flippable spin to align with the transverse field, assuming symmetric superposition and giving an energetic contribution proportional to the transverse field. This leads to a sixfold degenerate perturbative ground state. **b,c**, Each of these ground states maps to a complex pseudospin. **d**, A perturbative ground state for a single plaquette can be tiled across the lattice, maximizing the number of flippable states. **e**, When there is a fluctuation in the plaquette pseudospin field, triangles at the interface have no contribution from the transverse field, leading to an excitation proportional to the interface boundary and the transverse field.

cylindrical boundary, this is equal to the complex order parameter ψ as also defined in Equation (2) in the main text for the square-octagonal lattice:

$$\psi = \frac{1}{|\mathcal{P}|}\sum_j \psi_j = \left(\frac{1}{|S_1|}\sum_{i\in S_1} \sigma_i^z + \frac{e^{i2\pi/3}}{|S_2|}\sum_{i\in S_2} \sigma_i^z + \frac{e^{i4\pi/3}}{|S_3|}\sum_{i\in S_3} \sigma_i^z \right) /\sqrt{3}. \tag{2}$$

Considering only states in which each triangle is in either a quantum or classical ground state, we note that since any two neighboring triangles share two spins, their pseudospins differ in angle by at most $\pi/3$. Indeed, starting from a perturbative ground state of the lattice, we can rotate a region by $\pi/3$ into a different perturbative ground

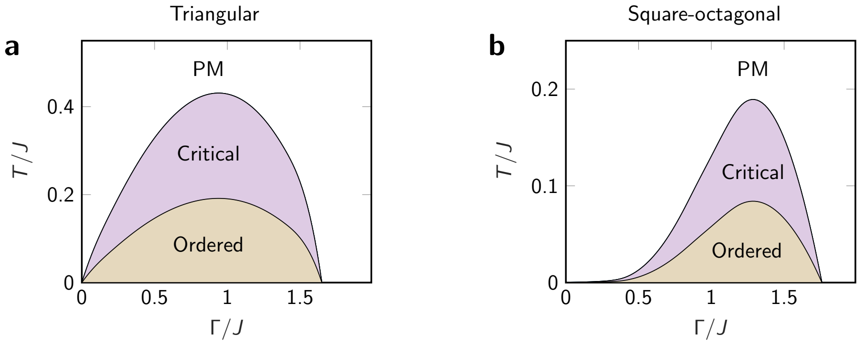

Supplementary FIG. 3. **Phase diagrams of triangular and square-octagonal lattices.** Both the triangular lattice (**a**) and the square-octagonal lattice (**b**) have rich phase diagrams with the same qualitative features: In the Γ/J, T/J plane, ordered and critical regions form domes delineated by topological Kosterlitz-Thouless phase transitions at finite temperature, with a quantum critical point at $\Gamma/J \approx 1.65$ and $\Gamma/J \approx 1.76$ respectively[2,5].

state. The excitation created by this fluctuation in the pseudospin field comes from its boundary: each triangle at the interface between the two phases is in a classical ground state with no energetic contribution from the transverse field. Thus the energy associated with rotating a domain by $\pi/3$ against a prevailing pseudospin alignment is linearly proportional to both the size of the fluctuation's boundary and the strength of the transverse field.

Although the microscopic details are different, this situation is qualitatively similar to the six-state discrete XY model[13] (sometimes called the six-state clock model[14]) used to describe certain properties of the phase diagram. In this model each degree of freedom is a rotor with angle $\theta_i = 0, \pi/3, 2\pi/3, ..., 5\pi/3$, with Hamiltonian

$$H = -J_{\mathrm{XY}} \sum_{\langle i,j \rangle} \cos(\theta_i - \theta_j). \tag{3}$$

In this model adjacent rotors can differ by more than $\pi/3$. The relationship between the discrete XY model and the triangular lattice was studied in the stacked magnet by Blankschtein et al.[9] and later established fully in the transverse field Ising model via theory and PIMC simulations[3–5]. In the limit $\Gamma \to 0$, the corresponding XY exchange strength J_{XY} is linearly proportional to the single-qubit tunneling Γ [8]. It is in the pseudospin that we see the familiar topological excitations—vortices and antivortices—that lead to topological phase transitions in the transverse field Ising model[2,4,15–17].

The work of Moessner and others[3–5] expanded the picture beyond the perturbative limit, establishing a rich phase diagram (Supplementary Fig. 3) in the $(\Gamma/J, T/J)$ plane with an ordered phase at low temperature forming a dome between the critical point at $\Gamma = 0$, $T = 0$ and a quantum critical point at $\Gamma/J \approx 1.65$, $T = 0$ [5]. Above the ordered phase there is an extended critical region with power-law decay of correlations, and at even higher temperature the system is paramagnetic. The finite-temperature phase transitions bounding the critical region are Kosterlitz-Thouless phase transitions in the 2D XY universality class, while the quantum phase transition is in the 3D universality class[4].

b. *Square-octagonal lattice*

Having established the situation for the triangular antiferromagnet in the transverse field Ising model, we return to the subject of the experimental demonstration: the square-octagonal lattice. Qualitatively, the foundation we laid out for the triangular lattice transfers directly to the square-octagonal lattice. The classical ground states are in one-to-one correspondence between the square-octagonal and triangular lattices, since no ferromagnetic chain bond is frustrated in a ground state. Therefore both lattices are critical at $\Gamma = 0$, $T = 0$. More generally, it was previously established (see [2] Extended Data Fig. 9) that this lattice has a phase diagram with all the features seen in the triangular lattice, shown in Supplementary Fig. 3.

It should be recognized that for the temperature, transverse fields and lattice sizes studied herein finite size effects are strong, and behaviour cannot be well characterized by perturbative approximations or the thermodynamic limit. The phase diagram is a guide, and the perturbative limits provide some intuition, but we do not argue that the parameterizations studied (Fig. 3a) are exemplars for the phases predicted by a scaling analysis. Understanding deviations and corrections to the thermodynamic limit at mesoscopic scales is a task well suited to PIMC and QA relative to other methods that may capture only thermodynamic or perturbative limits, and methods that capture

only qualitative or universal features of the phases. Furthermore, the triangular lattice is not sufficient to understand the square-octagonal lattice physics.

One important difference from the triangular lattice is that in the perturbative limit $\Gamma \to 0$, order-by-disorder can be explained by energy splitting from cotunneling of a four-qubit chain in a Greenberger-Horne-Zeilinger (GHZ) state $(|\uparrow\uparrow\uparrow\uparrow\rangle + |\downarrow\downarrow\downarrow\downarrow\rangle)/\sqrt{2}$, rather than the tunneling of a single spin. This difference in the number of qubits involved in cotunneling explains the difference in the shape of the phase diagram at small Γ. The ordering in the square octagonal lattice also occurs at lower temperature in the triangular lattice, this can be intuitively understood by noting the additional degrees of freedom per plaquette. As a consequence of the increased entropy, disordered phases are preferred to lower temperature. Although there is a one-to-one correspondence between ground states of the triangular and square octagonal lattices, additional patterns of excitation are possible around plaquettes in the square octagonal case owing to frustration of ferromagnetic bonds. Such frustrations are common at the temperatures and transverse fields we study. Perhaps the most interesting consequence of such breaks is a symmetry breaking between directions of pseudospin rotation (Fig. 2e Supplementary Fig. 22). At finite temperature we can also demonstrate that patterns of entanglement between and within chains are distinct from those in the triangular model (Methods 8).

c. *Initial conditions for the simulation*

The QA processor can be initialized with an arbitrary classical Ising spin state. The simplest of these is an *ordered* state (Fig. 1d), in which the three sublattices are $\uparrow\uparrow\downarrow$ or $\downarrow\downarrow\uparrow$ in some permutation. There are six such states, and they have have complex order parameter $\psi = e^{ik\pi/3} \cdot 2/\sqrt{3}$, $k = 1, \ldots, 6$ (Fig. 1c). Accordingly, we denote the states by S_k, $k = 1, \ldots, 6$.

To construct the CCW initial condition for a lattice on $6m$ rows of four-spin chains (Supplementary Fig. 4), we divide the lattice into six slices, with slice k being the spins of rows $((k-1)m+1), \ldots, km$. On the spins of slice k, we assign the value they are given in S_k. In this way we construct a state in which the pseudospin winds in a full rotation along the periodic dimension of the cylinder. Similarly, we construct the CW initial condition by giving the spins of slice k the value they have in state S_{7-k}.

The CCW and CW states are thereby constructed with topological obstruction. They are classical ground states, and through four-qubit flips we can explore limited parts of the ground state manifold. It is impossible, however, to escape the topological winding: one cannot reach an ordered state with these local rearrangements. In this sense the CCW and CW states correspond to metastable valleys in the quantum potential when a perturbative transverse field is added.

Moreover, the CCW and CW states differ from one another in subtle ways. Since the four-qubit chains have an orientation with respect to the cylindrical boundary condition, they are not actually symmetric to one another, unlike the corresponding configurations in the triangular lattice. In terms of both equilibrium properties and escape dynamics, there are subtle differences between the CCW and CW states. The fact that these are captured in the QA simulation (Fig. 2e, Supplementary Fig. 22) is quite remarkable.

We study lattices on $4L(2L-6)$ spins with cylindrical boundary condition, with L chains from one open boundary to the other, and $2L-6$ chains around the periodic dimension. AFM couplings at the boundary are set to $J_{ij} = 1/2$ instead of 1 in order to maintain high degeneracy of classical ground states. We study instances with $L \in \{6, 9, 12, 15\}$, the largest system having 1440 spins. The presence of inoperable qubits prevents us from repeating the study of an 1800-spin system[2]. For each lattice size we use two embeddings (Supplementary Fig. 5) to ensure consistency of results.

The construction of the square octagonal lattice for every lattice size studied, and construction of the classical initial condition, are implemented in C++ in the open source code (Methods 10).

d. *Winding number*

We quantify topological winding of the pseudospin field as follows. We take a pseudospin field $\mathcal{P}$ as a square lattice whose rows alternate between neighboring octagonal and square plaquettes; a cylindrical lattice with $L = 15$ made up of a 15-by-24 lattice of chains then becomes a 28-by-24 square grid of plaquette pseudospins. We then take the two-dimensional Fourier transform

$$M(a,b) = \sum_{j=0}^{L_1-1} \sum_{k=0}^{L_2-1} \exp(2\pi i \tfrac{ja}{m}) \exp(2\pi i \tfrac{kb}{n}) \mathcal{P}_{j,k} \tag{4}$$

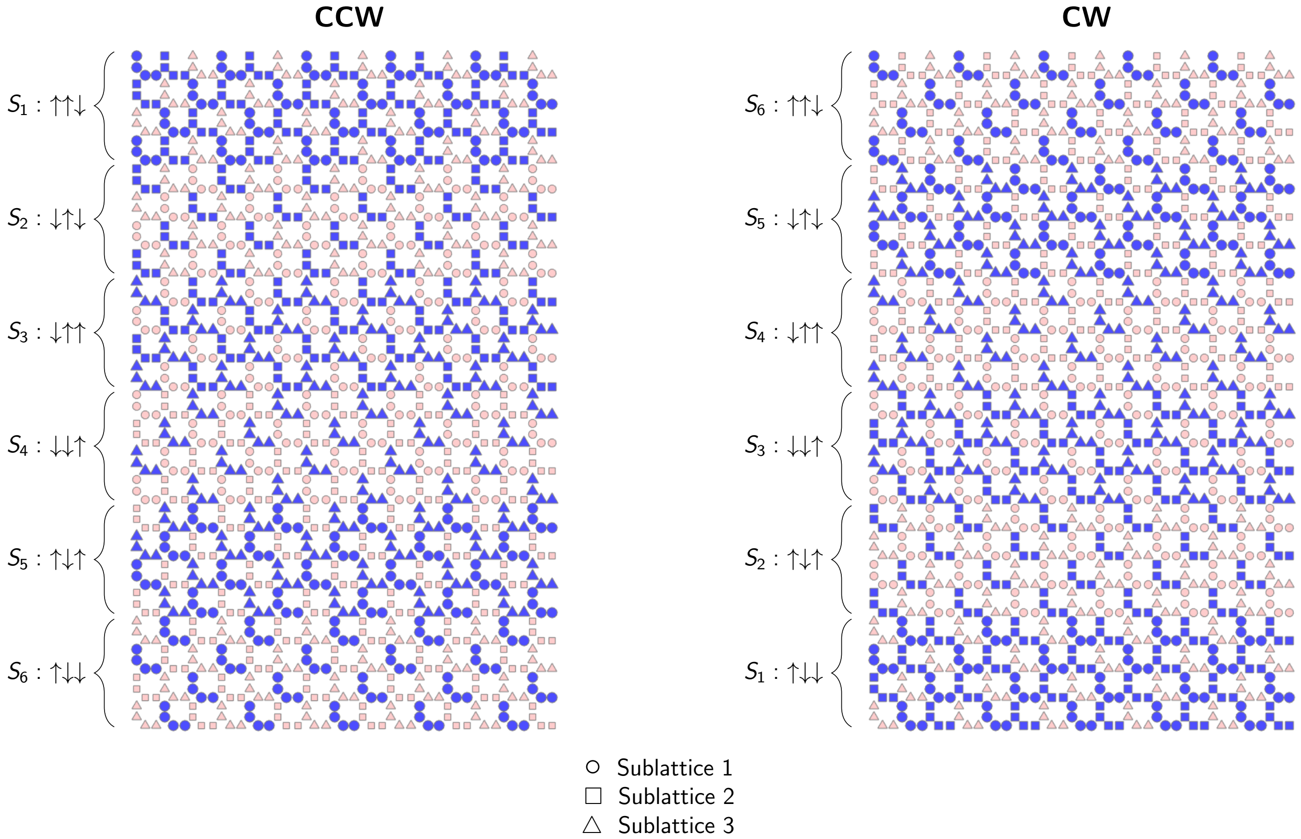

Supplementary FIG. 4. **CCW and CW wound initial conditions.** Shown are examples of wound initial conditions for the largest system size studied: a 24×15 lattice of four-qubit chains. The lattice is divided into six stripes of four rows each; each stripe is assigned to a different ordered initial condition S_k winding around in a complete rotation. The three sublattices, derived from the triangular antiferromagnet, are shown with different marks: circles, squares, and triangles respectively. The state S_k has complex order parameter $e^{ik\pi/3} \cdot 2/\sqrt{3}$. The resulting CCW and CW states are ground states of the classical potential. The winding of the pseudospin provides a topological obstruction from which QA and PIMC must escape.

where L_1 and L_2 are the dimensions of the square lattice representation of $\mathcal{P}$. Note that the zeroth order in the Fourier term is proportional to the order parameter up to corrections at the boundary $m = M(0,0)/(L_1 L_2) + O(1/L_2)$. For an integer winding number w, we define

$$f(w) = \sqrt{\sum_{a=0}^{L_1-1} \left(\frac{|M(a,w)|}{L_1 L_2} \right)^2}.$$

This gives us 24 possible values of w. The ordered, CCW, and CW states have peaks in $f(w)$ at $w = 0$, $w = 1$, and $w = -1$ respectively. The relationships between equilibrium values of these quantities is explored in Fig. 2e and Supplementary Fig. 22, offering a richer set of observables than just the order parameter.

2. Quantum annealing processor

Quantum annealing experiments were performed on a quantum processing unit with a similar circuit architecture to the processor used in previous quantum simulation experiments[1,2,18] but using a lower-noise fabrication process. In the previous system[2,18], all 2048 radio-frequency SQUID flux qubits and 6016 couplers were operational. In this experiment, 2030 qubits and 5909 couplers were operational; consequently we studied systems of up to 1440 spins instead of up to 1800 spins.

Each lattice size is embedded in two different ways in the qubit connectivity graph of the QA processor, differing primarily by a half rotation. As in previous experiments[2], we maintain a calibration refinement to compensate

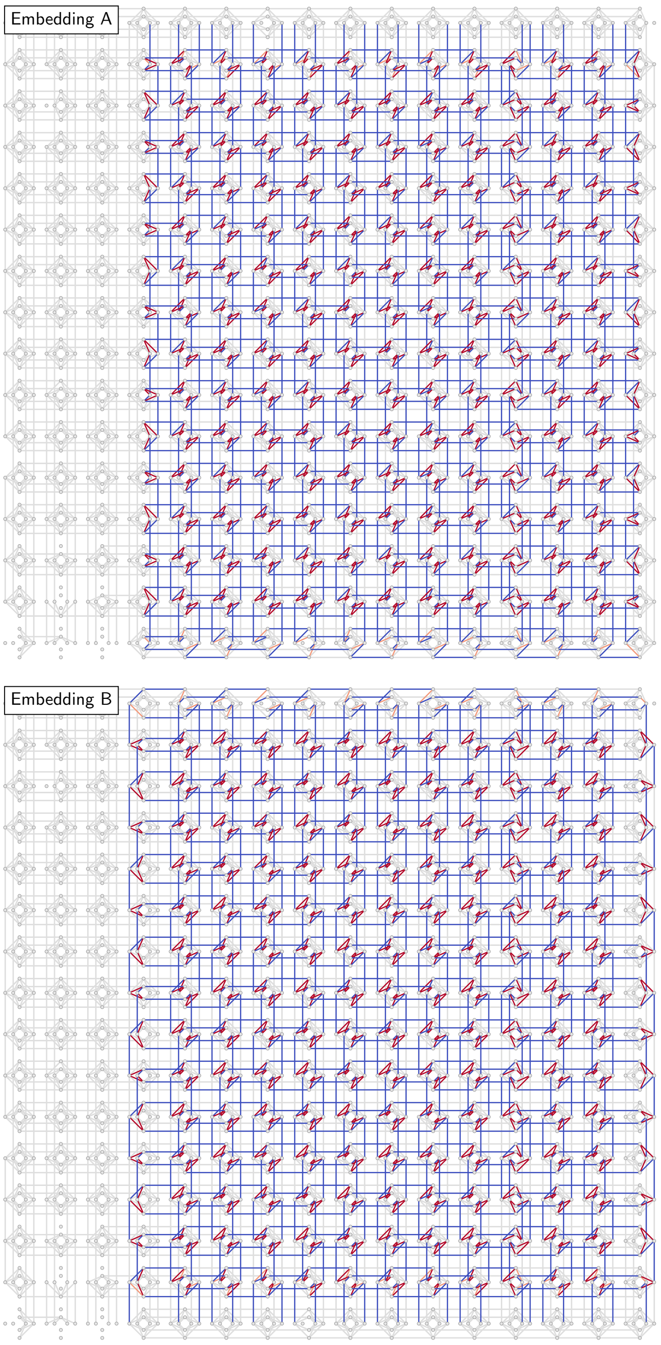

Supplementary FIG. 5. **Lattice embeddings for the 1440-spin system.** All experiments are run using two lattice embeddings to ensure consistency; they differ mainly by a rotation of 180 degrees. Shown here are the embeddings of the largest lattice simulated in this work, which has $L = 15$ chains from one end of the cylinder to the other, and $2L - 6 = 24$ chains around the periodic dimension.

for static crosstalks and minimize time-dependent drift in the Hamiltonian. This refinement minimizes variance in individual qubit magnetizations using flux-bias offsets. Using fine-grained modifications of coupling energies, it minimizes variance among the statistics of isomorphic antiferromagnetic couplers that relate to one another either by rotation of the cylinder or by isomorphic location in the two embeddings.

Quantum annealing itself typically involves sweeping the annealing parameter s from 0 to 1 in the Hamiltonian (Equation (1) in the main text), using a schedule of $J(s)$ and $\Gamma(s)$ such that $J(0) \ll \Gamma(0)$ and $J(1) \gg \Gamma(1)$ [19]. This approach is closely related to adiabatic quantum computing[20,21] performed at finite temperature. In this work we use a QA processor to probe Hamiltonian (Equation (1) in the main text) at an intermediate value of s, rather

than annealing s gradually. Experiments were run in a quantum evolution Monte Carlo (QEMC) loop[2], which mimics Markov-chain Monte Carlo but replaces the Markov chain update step with a reverse anneal. The QPU is initialized with a classical state, which for the first reverse anneal is a specified input, and for later anneals is the output of the previous step. Each anneal, aimed at evolution of the Hamiltonian $H(s^*)$, begins with $s = 1$, reduces s to s^* over $1-s^*$ microseconds, pauses for time t_p, then increases s from s^* to 1 over $1-s^*$ microseconds (detail in Supplementary Fig. 7). The major benefit of using QEMC in this experiment is that it allows fine-grained examination of intermediate points of the relaxation from initial condition to the converged distribution. At the end of each anneal, we insert a pause of 10 ms to minimize potential heating and sample-to-sample correlation. These pauses dominate overall experimental time and we have not attempted to optimize them as a parameter. Experiments were run for a range of temperatures, annealing parameters, and lattice sizes. Estimates of dynamics, with the exception of Fig. 3d for $s \leq 0.35$, were drawn from 300 QEMC chains of 16 samples each, for each of two lattice embeddings. Equilibrium estimates, and data for $s \leq 0.35$ in Fig. 3d, were drawn from 60 chains of 128 samples; for equilibrium estimates we discard the first half of the chain as burn-in.

a. *Extracted QA timescales*

Since measured QA timescales are often near the minimum experiment time of 1 µs, we must determine a reasonable cutoff for measurements in which we have confidence. This goes hand in hand with our definition of convergence time. We fit observations at longer timescales to a simple exponential process

$$m(t) = (m_0 - m_f)e^{-t/\tau} + m_f \tag{5}$$

where m_0, m_f, and τ are fitting parameters. The long-time limit m_f is well defined as the equilibrium estimate of $\langle m \rangle$. Restricting our time to series data to longer timescales we capture predominantly a decay well approximated by a single exponent decay and sampling noise, even if many different timescales are relevant on shorter timescales. However, the exponent (τ) was found to be quite a high variance estimator. Therefore we define our convergence time to be the time at which the fit function converges to within a cutoff value of its equilibrium value, which correlates well with τ. The advantage of using a fit function relative to a non-parametric estimator for convergence to a cutoff is again to reduce noise in the estimator, particularly for faster convergences.

The choice of this cutoff is motivated by the quality of data, as illustrated in Supplementary Fig. 6. The chosen cutoff of 0.05 is significantly larger than variation of individual QA estimates of $m(t)$. This means that any measured QA convergence time of greater than 1 µs is supported by strong evidence. Reducing the cutoff could lead to the inclusion of extracted cutoff times with weak supporting evidence.

b. *Insensitivity to quench rate*

In this work we have measured QA relaxation time using only the relaxation pause portion of the QEMC protocol (Supplementary Fig. 7). To justify this approach, we provide evidence that relaxation outside the pause is negligible. We do so by showing that QA relaxation is insensitive to changes in the quench rate.

Since we observe that QA convergence time varies exponentially in s over the observable range of roughly $0.36 \leq s \leq 0.40$, we consider the following oversimplified but illustrative model of relaxation far from equilibrium. Let us momentarily consider relaxation as an s-dependent task that is performed at a rate proportional to $e^{-\alpha s}$, for α chosen such that the system relaxes twice as fast at $s = 0.38$ as at $s = 0.39$. Since the quench and reverse anneal portions of the QEMC protocol have $|ds/dt| = 1/(1\,\mu\text{s})$ in our experiments, and pause time $t_p \geq 1\,\mu\text{s}$, the total change in m during each of the reverse anneal and quench phases is less than 2% of the change in m during the pause.

This model is vastly oversimplified but roughly in line with our observations. To show experimentally that out-of-pause relaxation is negligible in our experiments, we perform a spot-check at $s = 0.38$, $T = 13.7\,\text{mK}$ with pause time $t_p = 1\,\mu\text{s}$. We use reverse anneal and quench rates of $|ds/dt| = 1/(1\,\mu\text{s})$, $1/(2\,\mu\text{s})$, and $1/(4\,\mu\text{s})$ (Supplementary Fig. 7). The consistency of order parameter convergence as a function of pause time indicates that out-of-pause relaxation is negligible.

3. Measurement of QA parameters

The comparison of QA and PIMC relaxation requires detailed measurement of the QA processor parameters, and mapping to the resulting parameters of the implemented TFIM. In particular, we require measurements of qubit body

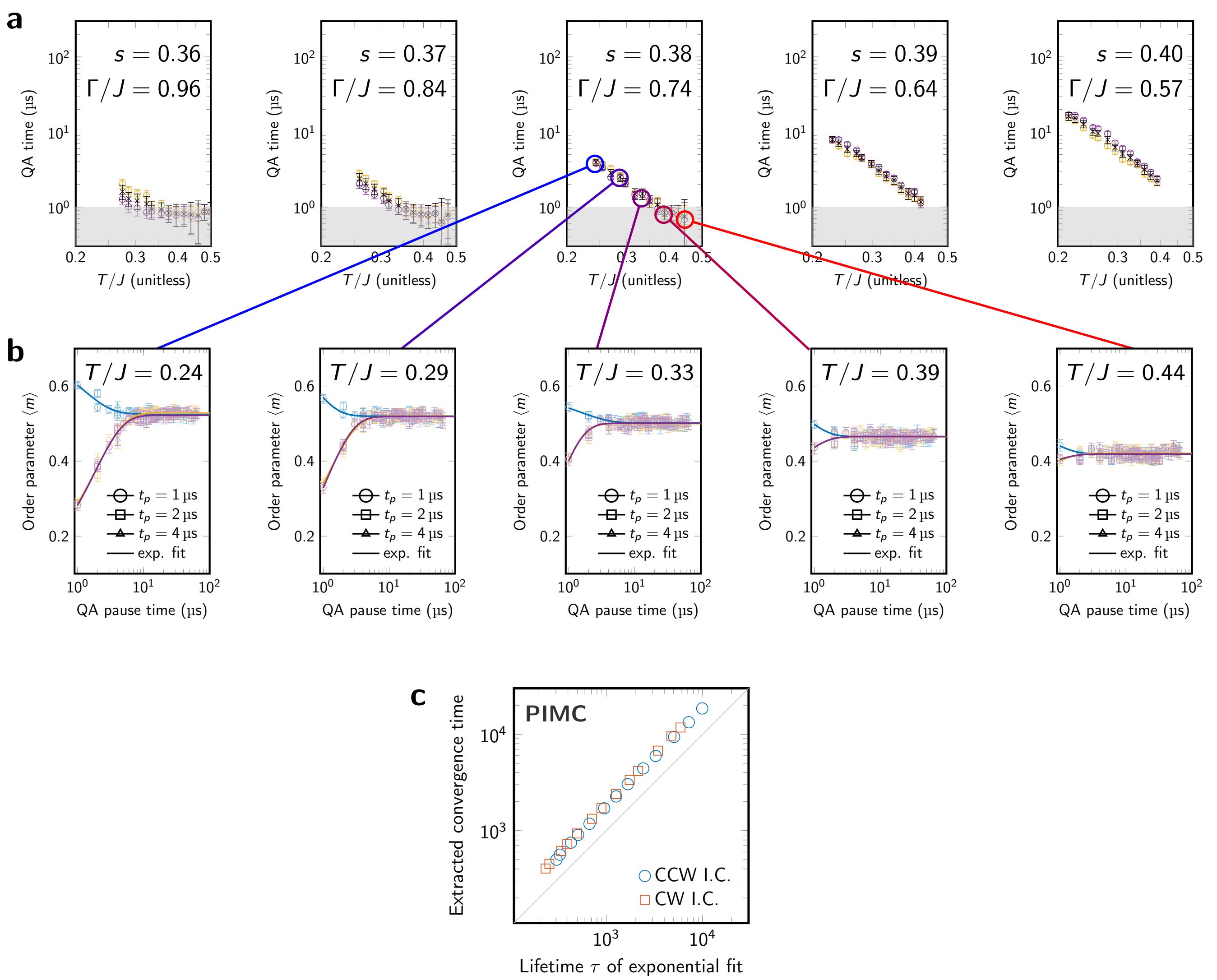

Supplementary FIG. 6. **Resolvable and unresolvable convergence times.** We consider any convergence times, given as the time at which the exponential fit function is 0.05 away from its terminal value, to be resolved only if it is greater than 1 µs. This condition is only relevant for QA, since all PIMC times are resolvable. **a**, QA times are extracted from exponential fits for a variety of annealing parameters s and temperatures T/J. Yellow and purple marks are from CCW and CW initial conditions, respectively; black marks are the geometric mean of the two, which we use as a single representative timescale. We discard any data for which either CCW or CW convergence time is less than 1 µs. **b**, Timescales are extracted from exponential fits to data from CCW and CW initial conditions. In the middle panel, for each of CCW and CW only three data points are significantly separated from the terminal value: 1×1 µs, 1×2 µs, and 2×1 µs (1×2 µs and 2×1 µs match closely, indicating that our measurement of relaxation time is accurate). These data are supported by the fact that convergence varies smoothly as a function of temperature, but for higher temperatures even the first data point is indistinguishable from sampling error, thus we cannot resolve a credible convergence timescale. **c**, Our extracted timescales, given by time to reach a cutoff of 0.05 in the exponential fit, is motivated by assuring statistical significance of the fit itself for small QA times. Here we scatter PIMC convergence times, extracted with the same methods, against lifetime τ of the exponential fit, showing strong agreement for $s = 0.38$ over a variety of temperatures. All error bars are 95% confidence interval on the mean.

inductance L_q, capacitance C_q, critical current I_c, and the two terms in the quantum annealing Hamiltonian: qubit tunneling energy $\Gamma_q(s)$, and qubit coupling energy $J_q(s)$, for the range of s we studied.

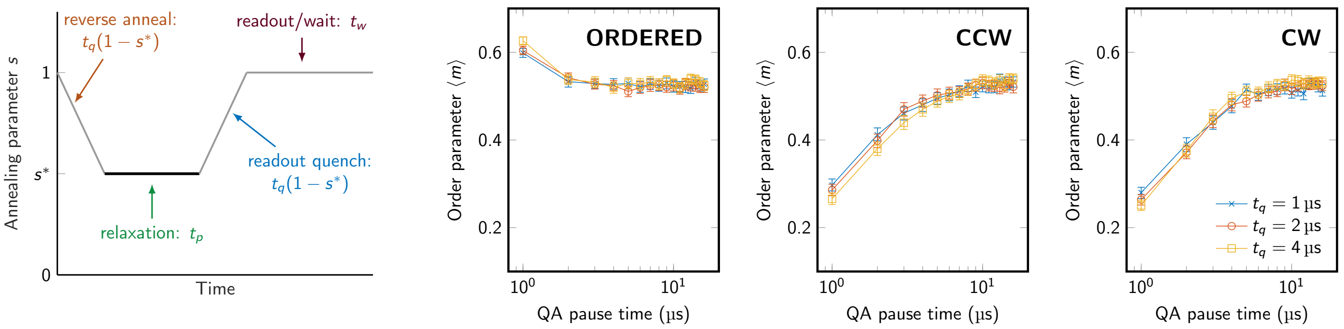

Supplementary FIG. 7. **Insensitivity to quench rate.** To support the claim that relaxation during quench and reverse anneal phases is insignificant, and therefore this time should be disregarded in analysis, we double and quadruple the amount of time spent during these operations. A systematic trend of "faster" relaxation for longer quench time would suggest that out-of-pause relaxation is significant; we observe no such effect. Data shown are for $t_p = 1\,\mu$s, $s = 0.38$, $T = 13.7\,$mK as in Fig. 2. Time spent during each of the reverse anneal and quench phases is $t_q(1-s^*)$, in this case $0.62\,\mu$s, $1.24\,\mu$s, $2.48\,\mu$s. All error bars are 95% confidence interval on the mean.

a. *Qubit model parameters*

We measure L_q and I_c by measuring qubit persistent current as a function of s in the the regime $\Gamma_q \ll J$ and fit to a classical model of the radio-frequency (rf) SQUID[22]. We measure $L_q = 284$ pH and $I_c = 2.34$ μA.

We then measure $\Gamma_q(s)$ and device capacitance with qubit tunneling spectroscopy. For a given value of s, we perform single qubit spectroscopy with a single attached probe qubit as described in Ref. 23. At this value of s, we sweep the qubit through degeneracy and fit the energy eigenspectrum to a one-dimensional dispersion relation. This gives a measurement of $\Gamma_q(s)$ and qubit persistent current, $I_p^q(s)$. We perform these measurements on 500 qubits for a range of s and fit the resulting data to a SQUID model to obtain a best fit capacitance of $C_q = 113.7\,$fF. Supplementary Fig. 9a shows these measurements along with the best fit to a SQUID model.

Finally, we calibrate $J_q(s)$ by performing qubit tunneling spectroscopy on coupled two-qubit systems. When two qubits are coupled with a term $J_q(s)\sigma_1^z\sigma_2^z$, then $E_2(s) - E_1(s) \equiv 2J_q(s)$ where $E_1(s)$ and $E_2(s)$ are the first and second excited state eigenenergies of the two-qubit system, respectively, at s. Note that this holds for all values of s, even when $\Gamma_q \approx J_q$. To perform spectroscopy, we choose a value of s and attach a third probe qubit to one of the two coupled qubits. We then measure the energy eigenspectrum of the system at degeneracy as described in Ref. 24. We measure 32 two-qubit pairs at a range of s to estimate $J_q(s)$ versus s. Supplementary Fig. 9b shows these measurements. We also show the prediction for $J_q(s)$ from the SQUID model. We emphasize that the dashed line in Supplementary Fig. 9b is not a fit: we use the calibrated device parameters and obtain excellent agreement between the two-qubit spectroscopy and the predictions from the SQUID model.

The square-octagonal lattice studied in this work contains sets of four qubits connected with $J_{ij} = -1.8$. The standard calibration we describe above is typically done with qubits coupled with $J_{ij} \approx 1$. Changing J_{ij} on couplers attached to a particular qubit causes an inductive loading shift which we compensate with an inductance tuner attached to the qubit[22]. We identified a small but systematic offset in the inductance compensation when tuning couplers to the strong ferromagnetic regime used in this study. We quantify this offset by repeating the calibration of L_q as described above, but with a coupler attached to the qubit tuned to $J_{ij} = -1.8$ rather than the typical $J_{ij} \approx 1$ we use. For the embedding used in this study, we measure an average change in qubit inductance of $\sim 1\%$. Specifically, the average qubit inductance increases by 2.9 ± 0.29 pH. Taking two standard deviations in this measurement gives a 95% confidence interval on the qubit body inductance of $L_q = 286.9 \pm 0.58$ pH; this uncertainty is a leading source of error in the QA schedule.

b. *Low Noise Processor*

We recently modified our fabrication stack to produce D-Wave 2000Q processors that have significantly lower flux noise. Supplementary Fig. 8 shows macroscopic resonant tunneling measurements on qubits from our standard fabrication stack and our lower noise fabrication stack. The macroscopic resonant tunneling protocol is described in detail here[18]. The broadening of the tunneling lineshape, W, is determined by the integrated flux noise experienced by the qubit during the tunneling protocol. For devices manufactured in our standard fabrication stack, measure an

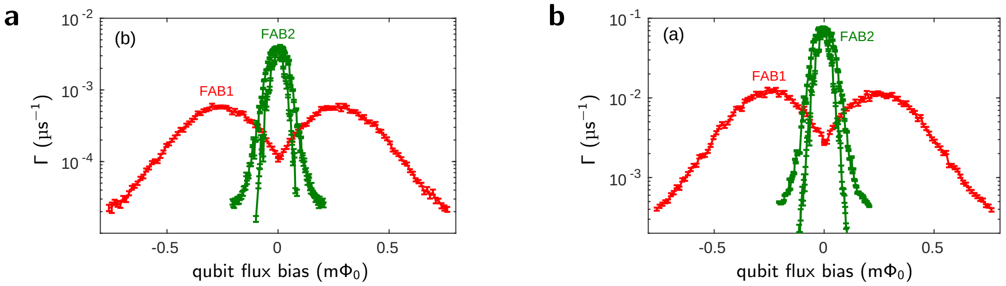

Supplementary FIG. 8. Single qubit macroscopic resonant tunneling measurement results for a standard D-Wave 2000Q processor (FAB1, labeled in red) compared to the low noise D-Wave 2000Q processor used in this study (FAB2, labeled in green) for similar tunneling amplitudes (a) $\Delta/h = 6.5$ to 7.0 MHz and (b) $\Delta/h = 1.5$ to 1.6 MHz. Two lobes represent the two possible initial states of the qubit ($\Gamma_{0\to1}$ and $\Gamma_{1\to0}$). Measurements are described in detail here[18]

integrated noise of $W = 145 \pm 2\ \mu\Phi_0$. For devices manufactured in our lower noise stack we measure an integrated flux noise of $W = 33 \pm 0.6\ \mu\Phi_0$.

c. *Qubit temperature*

We measure effective qubit temperature using a single-qubit susceptibility measurement described in detail in the Supplemental Material (p.8) of [25]. Equilibrium qubit population is measured for a range of biases to a qubit at degeneracy; these populations are then fit as a function of bias to a hyperbolic tangent (tanh) curve corresponding to a thermal distribution. From this fit we extract a qubit temperature. We measure the qubit temperature for all qubits used in the study. The measured temperature systematically deviates from the nominal cryostat set point by roughly one millikelvin. Supplementary Fig. 9c shows the relationship between nominal cryostat temperature and measured qubit temperature. Per-qubit variation in effective temperature is approximately Gaussian with a standard deviation of roughly 0.5 mK. Error in the mean qubit temperature of roughly 2% is estimated by taking the minimum and maximum over four independent temperature measurements.

d. *Background susceptibility and compensation*

A leading systematic deviation between our flux qubits and ideal spin-1/2 Ising moments is *background susceptibility* χ, through which qubits mediate a next-nearest-neighbor coupling. We estimate χ at a particular annealing parameter s by measuring deviation from ideal in the phase diagram of two coupled qubits under independent fields h_1 and h_2 (Supplementary Fig. 9d). As in previous experiments (see[2] Methods), we compensate for this behavior by tuning the programmed QA input couplings J_{ij} such that upon application of background susceptibility, the effective Hamiltonian approximates the desired square-octagonal TFIM. More formally, we denote the application of a background susceptibility χ to a classical Ising Hamiltonian H by $f_\chi(H)$. Using an iterative method, we find a Hamiltonian $H_{(-\chi)}$ such that $f_\chi(H_{(-\chi)}) \approx H$, as described in the Methods of previous work[2]. The relative tuning between AFM couplers is up to 2%, and the relative tuning between FM couplers is up to 9%.

e. *Effective spin-1/2 Hamiltonian*

The QA processor approximately implements two-level spin-1/2 qubits in the TFIM using rf-SQUID flux qubits, which have more than two energy levels. We have described measurement of the SQUID parameters. To determine and validate an effective spin-1/2 Hamiltonian for the fully-frustrated square-octagonal lattice, we diagonalize the SQUID Hamiltonian for a 12-qubit square-octagonal system (three chains) with periodic boundaries (Supplementary Fig. 10a). This gadget is used because it captures the three-sublattice ordering of the square-octagonal lattice, and is small enough to diagonalize easily. It also captures local signatures of entanglement in agreement with measurements over a larger lattice (Sec. 8). Since background susceptibility is included in the SQUID model, we diagonalize $H_{(-\chi)}$ instead of H. We perform approximate diagonalization using six energy levels per SQUID and retain 16 energy levels per four-SQUID chain. We find best-fit values of $\Gamma(s)$ and $J(s)$ so that the first eight eigengaps of the Ising Hamiltonian approximately match the first eight eigengaps of the SQUID Hamiltonian (Supplementary Fig. 10b).

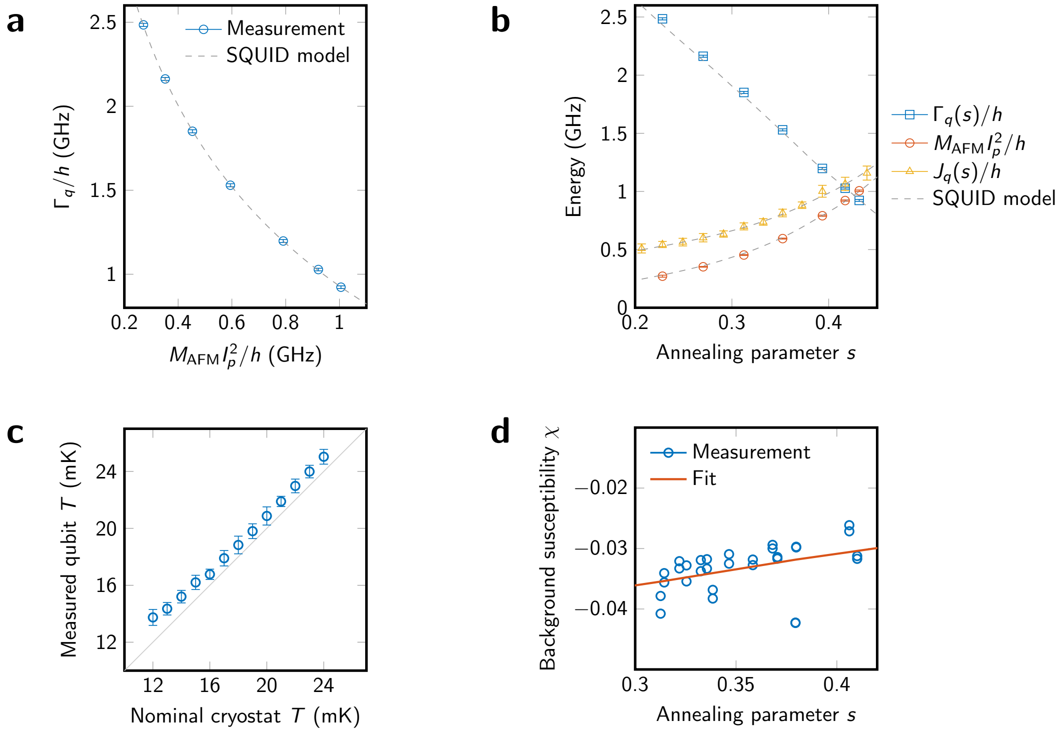

Supplementary FIG. 9. **Measurement of QA parameters.** **a**, Measurements of $\Gamma(s)$ versus $M_{\mathrm{AFM}} I_p^2$. The solid lines show the best fit SQUID model. **b**, Measurements of $\Gamma(s)$, $M_{\mathrm{AFM}} I_p^2$, and $J(s)$ versus s. The solid and dashed lines show the SQUID model. **c**, QA experiments are performed using nominal cryostat temperatures of between 12 mK and 24 mK. At each nominal temperature, an effective qubit temperature is measured via qubit susceptibility measurements. Error bars indicate uncertainty in mean qubit temperature, taken from the minimum and maximum of four independent measurements at each nominal temperature. **d**, Background susceptibility χ is measured through the study of two-qubit phase diagrams. We use linear regression values for compensation across experimental values of annealing parameter s.

The resulting parameters admit a mapping from the QA annealing parameter s to Ising parameters $\Gamma(s)$ and $J(s)$ (Supplementary Fig. 10c) with strong agreement between the low-energy spacing of the TFIM spectrum and the SQUID spectrum.

The main uncertainties in the QA schedule come from temperature T and qubit body inductance L_q. Supplementary Fig. 10d shows the QA schedule for several temperatures in the $(\Gamma/J, T/J)$ plane. The uncertainty in average qubit temperature gives a vertical error bar (hidden by markers) and the uncertainty in average qubit body inductance (± 0.058 pH) gives a diagonal error bar corresponding approximately to an uncertainty in s of ± 0.007.

4. Path-integral Monte Carlo methods

In this work we study several variants of path-integral Monte Carlo, a standard tool for estimating equilibrium statistics of systems in the transverse field Ising model. To estimate statistics accurately PIMC acts in the limit of continuous imaginary time (CT-PIMC), and we focus on this continuous-time form in the main body of the paper. While general-purpose PIMC code typically updates one spin at a time, collective tunneling of four-qubit chains is essential to the behavior of the square-octagonal lattice under study. It is therefore natural that PIMC simulation can be accelerated by collectively updating four-qubit ferromagnetically-coupled chains. We do so using Swendsen-Wang updates over four-qubit chains.

As with QA experiments, PIMC estimates are drawn from 600 independent repetitions for each parameter set. Raw output is projected to the classical space by taking the top Trotter slice. We perform up to 2^{22} PIMC4q sweeps to estimate equilibrium properties and convergence.

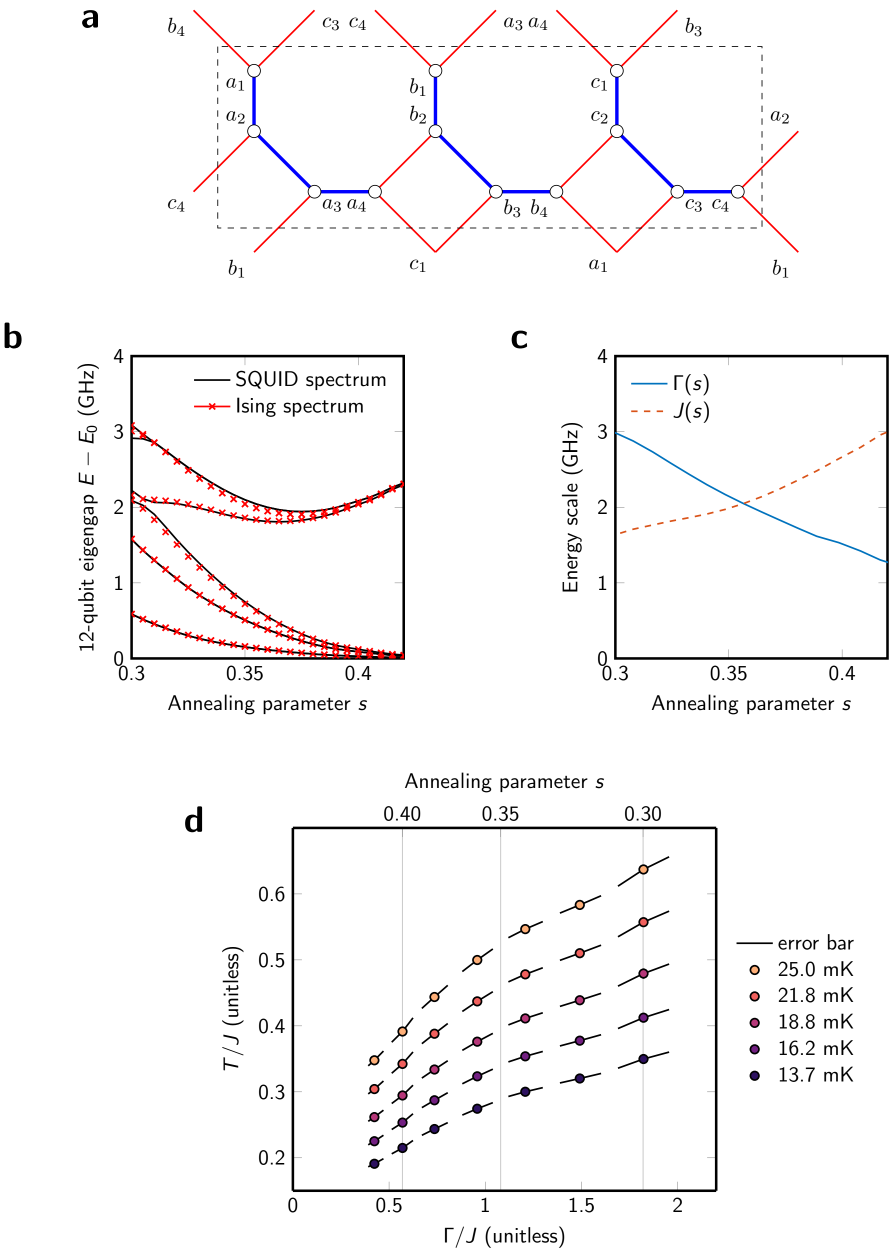

Supplementary FIG. 10. **Effective transverse field Ising model in a network of SQUIDs.** **a**, We study the spectrum of a minimal representative system: a 12-qubit square-octagonal lattice with periodic boundary. The QA processor uses rf-SQUID flux qubits to implement an effective transverse field Ising model. **b**, The lowest 9 energy levels of the SQUID spectrum are shown (some are degenerate), along with the spectrum of an effective Ising model whose parameters $\Gamma(s)$ and $J(s)$ are determined by fitting a weighted average of SQUID eigengaps. **c**, The parameters $\Gamma(s)$ and $J(s)$ provide the annealing schedule of the effective Ising model as implemented in flux qubits. **d**, Extracted Ising schedule is shown in the $(T/J, \Gamma/J)$ plane for a range of operating temperatures. The two major uncertainties are in the average qubit body inductance and the average qubit temperature, which respectively give diagonal and vertical error bars (vertical bars are hidden by the markers).

a. *Discrete-time PIMC and Trotter error*

The continuous-time PIMC approximation is based upon the Suzuki-Trotter transformation[26]

$$Z = \mathrm{Tr}\left[\exp\left(-\frac{J}{T}\hat{H}_P + \frac{\Gamma}{T}\sum_i \sigma_i^x\right)\right] = \mathrm{Tr}\left[\exp\left(-\frac{J}{TM}\hat{H}_P\right)\exp\left(\frac{\Gamma}{TM}\sum_i \sigma_i^x\right)\right]^M + O\left(\left[\frac{\Gamma}{TM}\right]^2\right) \qquad (6)$$

Where H_P is in this paper a diagonal matrix, σ^x are Pauli operators, and M is the number of *Trotter slices* into which the imaginary time dimension is discretized. We can associate the trace of the transformed quantity on the right to a sum over classical states of dimension $N \times M$ (space by imaginary time), called world-lines. For the TFIM there is no *sign problem*—i.e., the terms have positive weights—thereby allowing a Markov chain Monte Carlo (MCMC) integration[27–29]. The MCMC updates can be interpreted as a non-physical dynamics that is nevertheless known to capture correctly scaling in classical dynamics, and some special types of scaling in physical quantum dynamics[30]. Specialized hardware based on room-temperature nanomagnetic p-bits has even been proposed as a (manifestly classical) physical implementation of the discrete-time PIMC dynamics[31].

The associated Trotter error of discrete-time PIMC (DT-PIMC) can be driven to zero by making MT/Γ much larger than 1; in our CT-PIMC simulations we have taken M to be 2^{16}, large enough to reflect the infinite limit over our parameter ranges. However, one can compromise between Trotter error and convenience of algorithmic implementation; values of $M = 32$ or 64 are particularly convenient for bit-packing of the state into standard containers for which fast machine instructions exist, and allow for implementations on bandwidth limited platforms like GPUs. The methods we study all exploit the Swendsen-Wang algorithm over small qubit sets, though it is worth noting that for very small MT/Γ other non-cluster methods may become feasible in principle. In Supplementary Fig. 11 we show DT-PIMC estimates of $\langle m \rangle$ as a function of the number of Trotter slices M. Both convergence and equilibrium values are affected by the choice of M for parameters within our experimental range.

b. PIMC timing

Supplementary Fig. 12 shows timings of several variants of PIMC on a single CPU thread. For each variant, sweep time is dependent on T/J, Γ/J, and system size. Since time per MC sweep grows approximately linearly in the number of spins, we report only times for the largest system ($L = 15$, 1440 spins). Time per sweep varies as a function of T/J and Γ/J; we show the warmest and coldest QA temperatures over a range of Γ/J corresponding to annealing parameter s between 0.30 and 0.40. CT-PIMC and DT-PIMC code was run single-threaded on two comparable CPUs: CT-PIMC on an Intel(R) Xeon(R) CPU E5-2690 v3 and DT-PIMC on an Intel(R) Xeon(R) Platinum 8275CL, respectively.

We also measured timings for 32-slice, four-qubit-update DT-PIMC on an NVIDIA Tesla V100 GPU. For the 1440-spin lattice, running a single experiment gives a per-sweep time of roughly 40 µs independent of T and Γ/J. The experiment can be run on a GPU with a tradeoff between latency and throughput; optimizing for total throughput increases latency to roughly 50 µs per sweep, but decreases the total time needed for a throughput orientated experiment by a factor of roughly 120. A throughput orientated experiment may involve drawing many independent samples, or studying many different parameterizations, as we do here. This means that for an appropriately structured experiment, the high-end GPU can provide results approximately 1000 times faster than a single CPU thread for 32-slice DT-PIMC.

Supplementary Fig. 13 shows PIMC convergence time as a function of QA convergence time, as in Fig. 4, in units of both PIMC sweeps and PIMC CPU time.

c. Wall-clock time and power usage

We have compared convergence timescales in QA and PIMC simulations with the aim of studying relaxation dynamics. To take more practical benchmarking perspective, one can consider the total time taken to generate QA output samples, which we have made little effort to optimize. The QEMC cycle is dominated by a 10 ms wait time between consecutive samples; this parameter was chosen conservatively to allow settling of the system and to minimize heating. A spot-check indicated that reducing this to 2 ms resulted in no statistically significant difference in behavior. The next dominant factors are a programming time of 10 ms (amortized over the number of samples drawn) and a per-sample readout time of 274 µs. As a result of the wait time, the wall-clock time of the QA processor is roughly 10^4 times longer than the relaxation time under study. Removing the wait time and drawing many samples per programming leaves the QA duty cycle dominated by readout time, which in the case of $t_p = 4$ µs renders the wall-clock time roughly 10^2 times longer than the relaxation time. Since the focus of the study was relaxation dynamics rather than optimization of wall-clock time, we have not probed the effect of further reducing the wait time.

One important consideration in the utility of quantum processors is energy consumption. The QPU apparatus draws 25 KW, dominated by refrigeration. The 8176 and E5-2690 CPUs draw 6 W and 11 W per core respectively;

The i7-8650U CPU has a power specification (TDP) of 15 W; we can estimate the power-per-core (4 cores) as 3.75 W. The V100 GPU has a power specification of 300 W and, as mentioned above, can be up to 1000 times faster than a CPU core for this particular application. Thus taking the maximum time-advantage of QA over a CPU thread

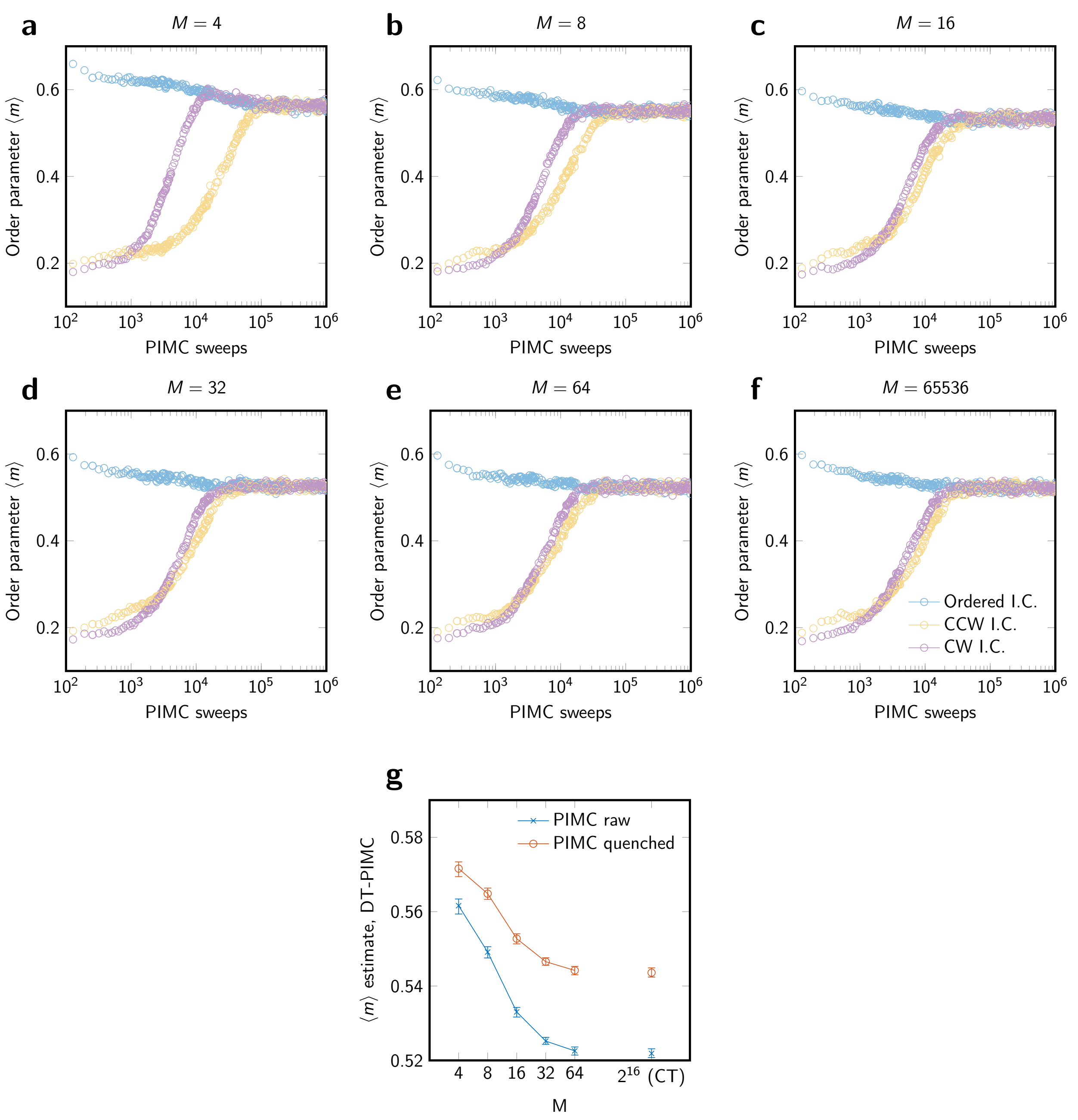

Supplementary FIG. 11. **Trotter error in discrete-time PIMC. a–f**, convergence of $\langle m \rangle$ estimates, analogous to Fig. 2b, for discrete-time PIMC with varying number of Trotter slices M, up to the continuous-time limit $M = 2^{16}$ (**f**). Model parameters are $\Gamma/J = 0.736$ and $T/J = 0.244$, with $L = 15$ (1440 spins). Imaginary time discretization has a strong effect on CCW/CW asymmetry in the lattice. **g**, varying M leads to varying estimates of order parameter $\langle m \rangle$ at equilibrium, for both raw and quenched PIMC samples. Estimates are taken from 2^{20} four-qubit update Monte Carlo sweeps; data points represent an average of 600 replicas from each initial state; error bars are 95% confidence intervals from bootstrap.

and an entire V100 GPU as approximately 10^6 and 10^3 respectively, we see that the quantum processor—counting only the relaxation pause time—can be over an order of magnitude more power-efficient than both CPU and GPU. If we consider QA wall-clock time, as discussed above, we see no power advantage. However, we expect the scaling of power requirements for near-term QA processors to show very flat scaling compared to computational power, since the dominant power draw—from a pulse-tube dilution refrigerator—is fairly constant.

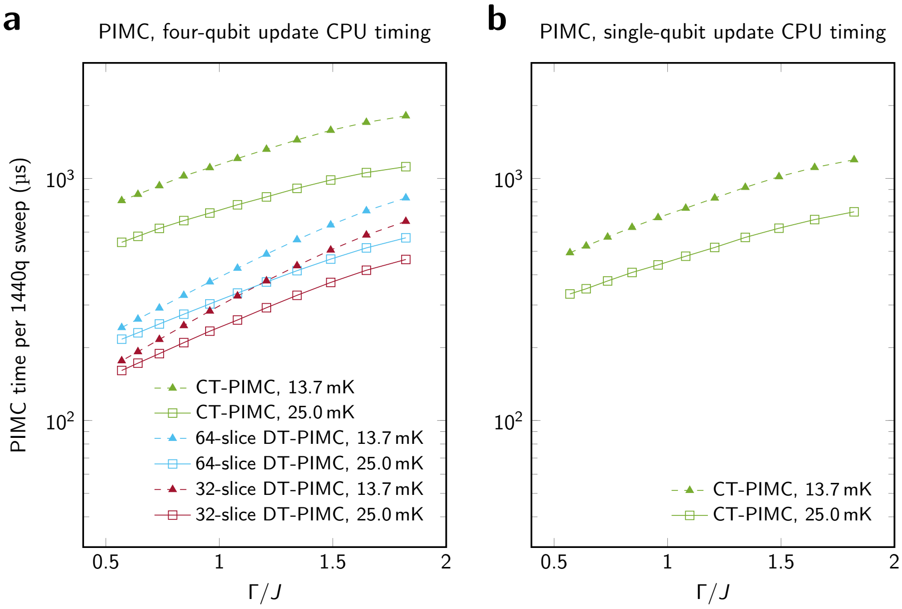

Supplementary FIG. 12. **PIMC timing.** Shown are average single-thread CPU time for a 1440-spin Monte Carlo sweep for **a**, four-qubit updates for continuous-time and discrete-time PIMC and **b**, single-qubit updates for continuous-time PIMC. Although single-qubit updates are faster in absolute terms, convergence in the square-octagonal requires many more single-qubit sweeps than four-qubit sweeps. Reported times in the paper use four-qubit continuous-time updates. Although time per sweep for a simple single-spin-update method (**b**) is faster than for four-qubit updates, convergence requires many more sweeps and hence the single-spin-update method is not competitive.

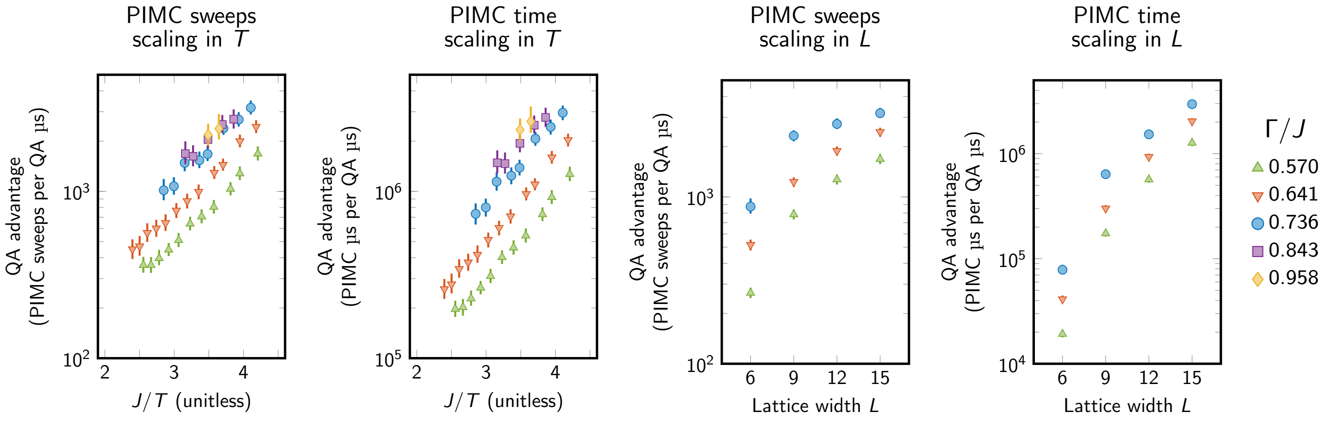

Supplementary FIG. 13. **Scaling of QA against PIMC.** Ratio of QA and PIMC time, measured either in PIMC sweeps or in CPU microseconds. All error bars are 95% confidence interval on the mean.

5. Competing methods: alternatives to PIMC

There are several methods one might consider using to estimate equilibrium statistics of the system in question. Here we lay out the case that PIMC is the most appropriate of these methods in this setting, justifying our focus on a comparison between PIMC and QA. It should be noted that it is impossible to explore all possible heuristic dynamics, and difficult to analyze results unrelated to physical dynamics or tailored with a-posteriori dynamical knowledge. The method we have chosen in the main text (PIMC) has a tight connection to physical dynamics, covers qualitatively a broad range of MCMC algorithms, and is considered standard in the field for reliable estimation of equilibrium statistics of finite-temperature TFIMs of dimension greater than one.

The most straightforward way to compute thermal expectation values of system observables is to diagonalize the Hamiltonian and compute a Boltzmann-weighted average over eigenstates. This is feasible for small systems, but

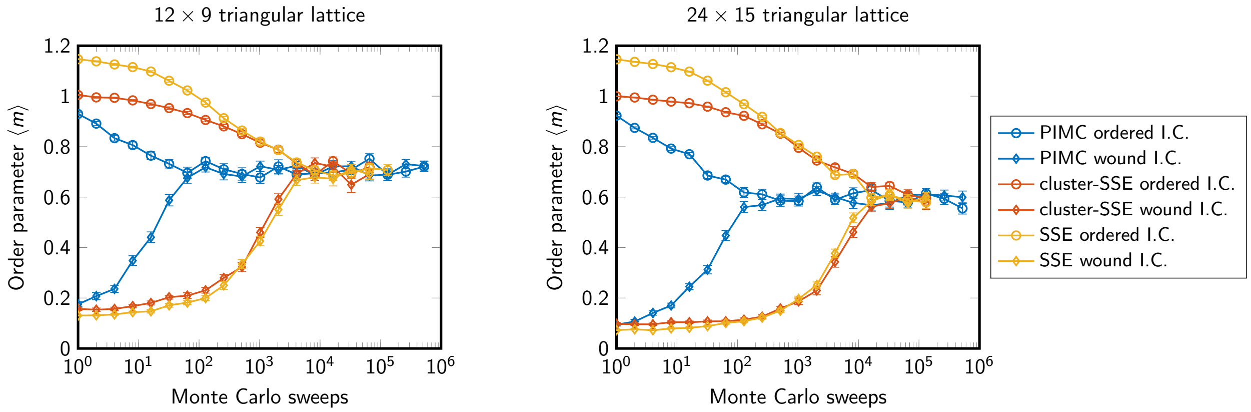

Supplementary FIG. 14. **Comparison of PIMC with SSE**. Shown is convergence of $\langle m \rangle$ averaged over 50 independent runs of PIMC, SSE and cluster-SSE from wound and ordered initial states on the triangular lattice (in this case CCW and CW are equivalent by symmetry). Simulations were run at $\Gamma/J = 0.6, T/J = 1/2$, which is qualitatively similar for the triangular lattice to the regimes studied in the square-octagonal case. Convergence measured in sweeps is significantly slower for both smaller (left), and larger (right), triangular lattices. Time per sweep is also found to be significantly slower in the SSE algorithms (see Table I). In the vicinity of the paramagnetic-to-critical transition we studied a number of parameters without finding a regime, or scaling, that would indicate a practical advantage to either cluster-SSE or SSE over PIMC. We anticipate a similar result in the more complicated square-octagonal case, for which modifications of cluster-SSE are required. All error bars are 95% confidence interval on the mean.

memory and time requirements scale exponentially. This makes the diagonalization approach infeasible around the 30-qubit scale for a laptop with only gigabytes of memory.

In some cases, especially one-dimensional chains, the size of the Hilbert space can be reduced, and a Lanczos diagonalization can be performed on the remaining core of the problem—this approach is called density matrix renormalization group (DMRG)[32]. Although DMRG initially applied to systems in the ground state (i.e., zero temperature), finite-temperature generalizations have been demonstrated[33], but the approach exploits the one-dimensional structure.

Similarly, tensor network approaches are extremely effective for 1D or quasi-1D systems, but are challenging to apply to the setting at hand. In fact, tensor networks have been applied to a geometrically-frustrated TFIM at finite temperature[17], by way of a quasi-1D transformation and application of an *exponential tensor renormalization group* (XTRG)[34]. However, this application was only successful up to the 108-spin scale, making it uncompetitive with PIMC in this context[17].

Diffusion quantum Monte Carlo (DQMC)[35] is a powerful method using Green's function to solve the Schrödinger equation, but only applies to the zero-temperature setting, not to the finite-temperature setting we explore in this work. Likewise, neural networks have been used for learning a many-body ground-state wavefunction[36], and although this method has recently been extended to the 1000-qubit scale[37], it has not been applied to statistical inference in large finite-temperature frustrated systems.

In Section 5 a we consider two standard Quantum Monte Carlo methods that are principled alternatives to PIMC. These methods are demonstrated not to accelerate equilibration compared to PIMC. The utility of these, and other, QMC methods in establishing equilibrium statistics is determined by transition rates over the phase space, particularly with respect to macroscopic dynamical barriers. It is non-trivial to design cluster algorithms to accelerate the most constraining dynamical barriers. Since the two algorithms studied exploit both a different representation of the state space, and in one case cluster moves tailored to fully frustrated lattices, they represent an interesting contrast to mechanisms present in spatially local PIMC approaches. These are only two of the many possible quantum Monte Carlo methods in existence; extensions to these QMC methods are possible through multi-canonical approaches (parallel tempering, simulated annealing, population annealing), or Markov chain heuristics breaking detailed balance.

a. Alternative MCMC methods: Cluster algorithms and stochastic series expansion

Stochastic series expansion (SSE)[29,38] is a Markov chain Monte Carlo framework distinct from PIMC. While PIMC uses the Suzuki-Trotter decomposition combined with spatially local moves, SSE works with a Taylor expansion of the Hamiltonian and spatially non-local cluster moves. SSE like PIMC has many successful applications, can have a superior scaling (factor N) relative to PIMC for some dense models, and has the advantage of not requiring a numerical

Supplementary TABLE I. i7 single core wall-clock time (seconds) for 2^{15} sweeps from CW initial state, seconds

Lattice dimensions	PIMC(C++)	SSE(C++)	SSE(MATLAB)	cluster-SSE(MATLAB)
12×9	1.2	21	7.0×10^3	4.7×10^3
24×15	4.0	78	7.5×10^4	5.4×10^4

approximation to a continuous time limit. The cluster-SSE algorithm we compare against introduces a variation on the SSE update suitable for AFM triangular lattices[39]. Since these algorithms are not spatially local, there is not an expectation to match physical dynamics even in the classical limit. However, these are exemplars for MCMC algorithms that achieve equilibrium whilst exploiting an algorithmic dynamics qualitatively distinct from spatially local dynamics in PIMC. Examining two forms of the SSE algorithm is not a demonstration that all alternative QMC algorithms will be slow, but shows that the slow equilibration of the order parameter is present under a variety of QMC principles, and that for practical purposes (in this model) PIMC is fastest.

The triangular lattice, aside from difference in local pattern of correlations and symmetry breaking in winding directions, has a qualitatively similar phase diagram and equilibration dynamics to the square octagonal lattice we study (see Supplementary Fig. 3 and related discussion). Since there exists a cluster algorithm (cluster-SSE) specifically tailored to the triangular lattice, it is interesting to compare against PIMC in the triangular space, also allowing the simplification of single-qubit moves. We can work at qualitatively similar parameters to those in our square-octagonal study near the paramagnetic-to-critical transition on cylindrical lattices. The comparison demonstrates that even in this simplified model, cluster moves (perhaps surprisingly) do not accelerate equilibration—at least, the slowest mode is qualitatively unchanged.

The state of the system in SSE is encoded in an operator sequence. We can define one sweep (iteration) of SSE, or cluster-SSE, as an update of all operator positions. We can use CW and ordered initial conditions, as for the PIMC case, and measure convergence time. As with the square-octagonal case these initial conditions trap dynamics for some timescale; we note that on the triangular lattice the CCW and CW wound states are symmetric, so the result for CCW is identical to CW and is not presented separately. Supplementary Fig. 14 shows the convergence pattern of all algorithms at representative parameters for two different lattice sizes. Measured in sweeps, there is no indication that SSE or cluster-SSE can accelerates convergence of the order parameter. For scaled fields $\beta\Gamma$ and βJ that are $O(1)$ we anticipate the time per sweep in efficient implementations to scale as $O(N)$ in SSE, cluster-SSE and PIMC, with some small variation as a function of other parameters, initial conditions and run length. This scaling of time per sweep can be inferred from the typical number of degrees of freedom operated over in one sweep (number of interfaces manipulated in PIMC, or number of operators updated in SSE). In Supplementary Fig. 14, we show sweeps to eliminate this known prefactor, in order to reveal any potential for scaling advantage and to remove implementation detail dependence (per sweep), but wall-clock times are important in a practical implementation. We measured wall-clock times for each algorithm on Intel i7 single core implementations; these values are shown in Table I. Our SSE implementations are significantly slower than the PIMC implementation. The C++ code use efficient implementations, but could be subject to further specialized optimizations that might close or expand the gap by a small factor. Our MATLAB implementations are not very efficient, and scale as $O(N^2)$, thus the reported cluster-SSE wall-clock time could be substantially reduced. However, by inspection of C++ versus MATLAB implementations of SSE, we can anticipate that an optimized implementation of cluster-SSE would remain much slower than PIMC per sweep, for this reason we have not made the effort to implement this algorithm since it is likely to be orders of magnitude too slow at relevant parameterizations.

We have reported in this table a value for PIMC based on our fully featured and optimized code, by contrast the open-source code is approximately 5 times slower (Section 10). Zealous optimization of the SSE C++ implementation might make a comparable gain, but we do not expect that we can make SSE or cluster-SSE faster per sweep than our PIMC implementations at these parameterizations.

The combination of weaker performance in time measured in sweeps, and in time per sweep operation, combines to make SSE and cluster-SSE inferior choices for the study of equilibrium. However, the situation is more nuanced: cluster-SSE is interesting in that it accelerates randomization of the phase θ of the order parameter $\psi = m \exp i\theta$, and this is achieved in only a few sweeps. In other experimental regimes we might expect randomization of the phase to be a slow process, in which case the cluster moves would be of greater utility, as concluded in the original paper[39]. Thus the cluster algorithm has some utility, but does not appear to accelerate equilibration as measured by the slowest and most interesting dynamical mode in our experiment.

The reason for failure of both SSE, cluster-SSE and any global cluster algorithm in the case of overcoming a topological obstruction is rather intuitive, and is supported by our empirical studies of SSE: Locally a wound state looks very much like an ordered state; the order parameter is approximately aligned up to a gradual phase rotation around some periodic lattice direction. A cluster algorithm, which grows clusters based on local information, cannot

effectively distinguish wound and unwound cases, and with high probability either selects a system spanning domain or a local domain. The cluster rules employed in SSE, cluster-SSE and similar methods rotate (or reflect) the entire pseudospin field uniformly. While this accelerates randomization of the phase, is does not contribute to unwinding. We can anticipate the same qualitative behaviour in PIMC large scale cluster moves.

6. Competing methods: simulation via approximate models

Geometrically frustrated two-dimensional transverse field Ising models are known to exhibit a number of interesting behaviors, not least the order-by-disorder phenomenon and competition between quantum and thermal fluctuations[3–5,11]. Interesting scaling has been exhibited in finite-size simulations of the square-octagonal and triangular lattices, attributed in part to a crossover with quantum critical phenomena[2,5]. A simple exact description of the perturbative limit (small T and small Γ) for the square-octagonal lattices demonstrates the importance of four-qubit GHZ states in the ground state[2]. In this paper we have examined the dynamics of unwinding; this is intuitively related to critical behavior in the form of system-spanning vortices. We have demonstrated results that indicate dynamical differentiation between dynamics of PIMC and physical QA dynamics. These various factors indicate the importance of quantum phenomena in describing the equilibrium properties of the lattice in question.

PIMC is a standard tool for simulating equilibrium properties of a finite-temperature TFIM. However, we want to confirm that we have compared QA against the best classical simulation in the specific setting of the square-octagonal lattice. Thus we need to rule out alternative approaches that could potentially simulate the square-octagonal lattice more efficiently than PIMC. The entanglement related to the GHZ states in the perturbative limit is localized. Near this limit, properties of the phase diagram can be explained without long-range quantum correlations. Furthermore the KT phase, being a finite-temperature critical phase, is known to have classical scaling: the scaling of extensive properties in system size near the critical point is consistent with classical models at large scale[40]. Finally, the low energy solution space seems to indicate the possibility for a reduction of the model from a square-octagonal to a triangular description by mapping four-qubit chains onto logical qubits, or an even simpler phenomenological model over plaquette pseudospins. Given these factors it is reasonable to ask if there are approximations to the square-octagonal transverse field Ising model that would allow for equally good approximation at lower cost, or expose the absence of quantum dynamics in accelerating unwinding.

Although it is impossible to disprove the existence of a superior simulation method for the lattice in question, in this section we rule out several reasonable approaches: quasi-classical approximations consistent with the absence of entanglement and quantum mechanisms, reductions of the square-octagonal model to a triangular lattice model, and a phenomenologically matched XY model. We have already demonstrated evidence for entanglement at short range, which places some restrictions on the quality of approximations achievable by quasi-classical approximations.

a. Six-state clock and other phenomenological approximations

One potential way to bypass long equilibration timescales in the lattice under study is to replace the TFIM with a simpler classical model exhibiting the correct phases and symmetries. The most natural candidate is the plaquette pseudospin, which accurately reflects the physics of the TFIM in the perturbative limit. A pseudospin describes the state of a frustrated plaquette, and takes one of six values in any classical ground state $\theta_i \in \{2\pi k/6 : k = 0, \ldots, 5\}$. These plaquette states are constrained through shared qubits; these constraints favor full or partial alignment of neighboring pseudospins. This interaction can be qualitatively captured by pairwise ferromagnetic couplings between six-state XY spins over a dual (honeycomb) lattice

$$H = -\sum_{ij} \cos(\theta_i - \theta_j),$$

where we can determine strength of coupling by the temperature T. The translation from the full model interaction to this simpler pairwise one is nontrivial at the microscopic level, but T can be tuned to optimize the approximation. The six-state clock model exhibits both a paramagnetic-to-critical transition and a low temperature crystalline state[13], and retains the symmetries of the square-octagonal model with respect to the classical ground states.

The TFIM phase diagram includes a quantum critical point at $\Gamma = \Gamma_c$; the clock model cannot reproduce the QPT or the domed phase diagram of the TFIM[2,5]. Still, suppose we wish to use the six-state clock model to approximate behavior in the vicinity of the paramagnetic-to-critical phase transition for some transverse field Γ with $0 < \Gamma < \Gamma_c$. The known critical scaling of both models dictates a divergence of the correlation length approaching the critical phase as $\xi \propto \exp(a/\sqrt{T - T_c})$. To correctly model the susceptibility, order parameter, and other extensive quantities—in

a scalable sense—it is necessary for this correlation length to be matched in both models. Knowing only the (non-universal) parameters a and T_c for each model we can require that the unitless correlation lengths match up to a prefactor. With this mapping between the model parameters in place, we can examine the approximation achieved by each at a given correlation length. This amounts to a comparison of the collapse form.

The six-state clock model on the hexagonal lattice can be considered an approximation to both the triangular antiferromagnet and the square-octagonal lattice. We first show deviations between the clock model and the triangular AFM via finite size scaling collapse, then show deviations between the triangular AFM and the square-octagonal model. To minimize finite size effects we work with periodic boundary conditions on $L \times L$ lattices. We fitted scaling collapse parameters using a smoothness condition, fixing the parameter c to 7/4, reflecting the 2D XY universal critical exponent of $\eta = 1/4$ (Ref. 5). Results are shown in Supplementary Fig. 15. The difference in forms is an indication that approaching the phase transition at equivalent correlation length there are substantial differences in susceptibility of the lattice, susceptibility growing more quickly in the TFIM and to a larger peak value at the phase transition—this is beside the Γ-dependence of TFIM scaling, which the six-state clock model cannot capture. The universal value leads to an excellent collapse of the 6-state clock model at these scales, but not so for our model due to more interesting finite size effects and a previously noted crossover impact from quantum critical phenomena[5]. Thus even after careful tuning of the parameters to match the growth of the correlation length, the predictive power of the pseudospin model for the TFIM is poor.

b. Triangular lattice transverse field Ising model approximation

There is a simple local one-to-one mapping between the classical ground states of the square octagonal model and those of the fully frustrated triangular model. The equivalence is realized in mapping the states of every four-qubit ferromagnetically-coupled cluster in the square octagonal lattice to a single qubit in the triangular lattice. By extension, the ground states in the presence of a perturbative transverse field are matched, where the single qubit superposition $(|\uparrow\rangle + |\downarrow\rangle)/\sqrt{2}$ is realized by a GHZ state over four qubits in the square octagonal case $(|\uparrow\uparrow\uparrow\uparrow\rangle + |\downarrow\downarrow\downarrow\downarrow\rangle)/\sqrt{2}$.

With this in mind one may expect that the triangular lattice can be used as a proxy for the square-octagonal lattice; this would require a mapping of Γ/J and T/J in square-octagonal lattice to Γ_t/J and T_t/J the triangular lattice such that the quantum critical points and finite-temperature phase transitions match. It would also require that the support of the square-octagonal wavefunction be dominated by chain-intact states $|\uparrow\uparrow\uparrow\uparrow\rangle$, $|\downarrow\downarrow\downarrow\downarrow\rangle$, or their superposition. However, even within the experimental parameters this is not the case, as evidenced by asymmetry between CCW and CW winding around the cylinder. These directions are symmetric in the triangular lattice, but not in the square-octagonal lattice; this subtlety is accurately simulated at equilibrium by QA, as shown in Supplementary Fig. 22c. The asymmetry is also clearly visible in the world-line dynamics of PIMC, as seen in convergence from CCW and CW initial conditions (Fig. 2b).

c. Spin vector model

The standard spin-vector rotor model associates angles $\theta \in [0, \pi]^N$ to qubits on the lattice, and has Hamiltonian

$$H(\theta) = \sum_{ij} J_{ij} \cos(\theta_i)\cos(\theta_j) + \sum_i h_i \cos(\theta_i) - \Gamma \sum_i \sin(\theta_i),$$

derived by replacing σ^z and σ^x in the TFIM Hamiltonian with $\cos(\theta)$ and $\sin(\theta)$ respectively. A spin vector Monte Carlo (SVMC, also SSSV after proponents[41]) was proposed as a semiclassical model that approximately reproduces QA performance in certain situations. Entanglement in a QA processor was later demonstrated experimentally[24] in agreement with the quantum adiabatic master equation and to the exclusion of SVMC[42]. While the spin-vector ground state can be identified with the ground state of a quantum system that is separable at the level of qubits, we have already demonstrated the presence of entanglement both within and between four-qubit chains. Since the ground state of a ferromagnetic chain in the rotor model under a perturbative transverse field is not aligned with the transverse field (this would be analogous to delocalized superposition of the chain), the rotor model cannot reproduce the low-temperature physics of the square-octagonal lattice. Furthermore the square-octagonal lattice has no quantum phase transition in the spin vector model, so any mapping between SVMC and PIMC in this setting will be approximate at best. As shown in Supplementary Fig. 18c–d, SVMC deviates significantly from both QA and PIMC in the range of parameters studied.

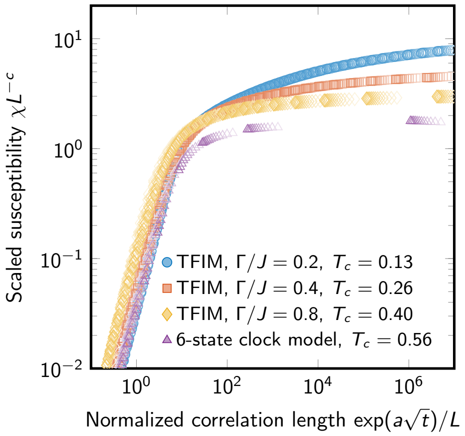

Supplementary FIG. 15. **Finite size scaling collapse of TFIM and pseudospin models.** We compare collapses of the triangular AFM TFIM at several values of Γ/J with the six-state clock model derived from the plaquette pseudospin. Scaling parameter c is clamped to the value 7/4 derived from 2D XY universality. Marker opacity indicates system size from $L = 9$ to $L = 36$. Differing scaling forms indicate that behavior of the fully-frustrated TFIM cannot be derived merely from critical temperature T_c, and that the classical six-state clock model fails to predict properties of the quantum system when reparameterized to match correlation length.

7. Effect of quench and disorder

Two important differences between the QA processor and an ideal system are effects of the readout quench, and analog misspecification in the Hamiltonian, e.g., device inhomogeneity. To understand the effect of this misspecification on statistical estimates, we run PIMC with static (quenched) disorder in the Hamiltonian. We perturb the classical Ising Hamiltonian by adding i.i.d. Gaussian terms with standard deviation $\sigma = 0.02$ to each linear term h_i and to each nonzero coupling term J_{ij}. We instantiate these errors independently for each sample, giving 600 instantiations of error for each initial condition. Results are shown in Supplementary Fig. 16. The perturbations in the Hamiltonian have no significant effect at high temperatures, but suppress $\langle m \rangle$ slightly at low temperatures; this is consistent with Fig. 2c, where we see QA estimates deviating slightly below quenched PIMC estimates at low temperatures. The analog error does not change convergence timescales significantly.

Supplementary Fig. 7 demonstrates that changing the length of the quench has little systematic impact on relaxation timescales. That is, although the total length of the quench and reverse anneal are similar to the pause time t_p, dynamics appear to be frozen almost immediately. However, this does not rule out the possibility that the act of repeatedly quenching and reverse annealing in QA distorts the measured timescales. Supplementary Fig. 17 shows QA data for several slow-converging models at $t_p \in \{1\,\mu s, 2\,\mu s, 4\,\mu s\}$. We see no evidence of systematic distortion of the data arising from the quench protocol.

We now consider effects of the readout quench on estimated equilibrium statistics. In this experiment the QA readout quench has been used as an approximation to projective readout. However, the system shows evidence of local relaxation during this quench; we estimate that the timescale of relevant dynamics is on the order of tens of nanoseconds. To address this issue, we take two approaches.

The first approach is to quench PIMC output, which we do by taking a PIMC spin state, projected to the σ^z basis, and applying a greedy descent in the classical potential: first we repair frustrated four-qubit chains by majority vote, breaking ties randomly. Next we greedily flip chains while doing so lowers the classical energy. This allows us to compare quenched PIMC output with quenched QA output; this is the approach we take in the main body of this work.

The second approach is to "unquench" QA output by applying a small number (10) of PIMC postprocessing sweeps. After this postprocessing we project the PIMC state to the σ^z basis as with our PIMC experiments; this allows us to compare unquenched QA output with projected PIMC output.

In Supplementary Fig. 18 we show the effect of this unquenching on the average order parameter and residual energy. While the order parameter is relatively robust to the process due to being somewhat topologically protected, the PIMC postprocessing sweeps dramatically increase the residual classical energy, which is dominated by local excitations (i.e., bound vortex-antivortex pairs). We see that raw QA output has systematically higher residual energy than quenched PIMC output; this is because excitations arising from frustrated ferromagnetic bonds are completely removed by the PIMC classical quench. We confirm in Supplementary Fig. 18e–h that our conclusions about advantage of QA over

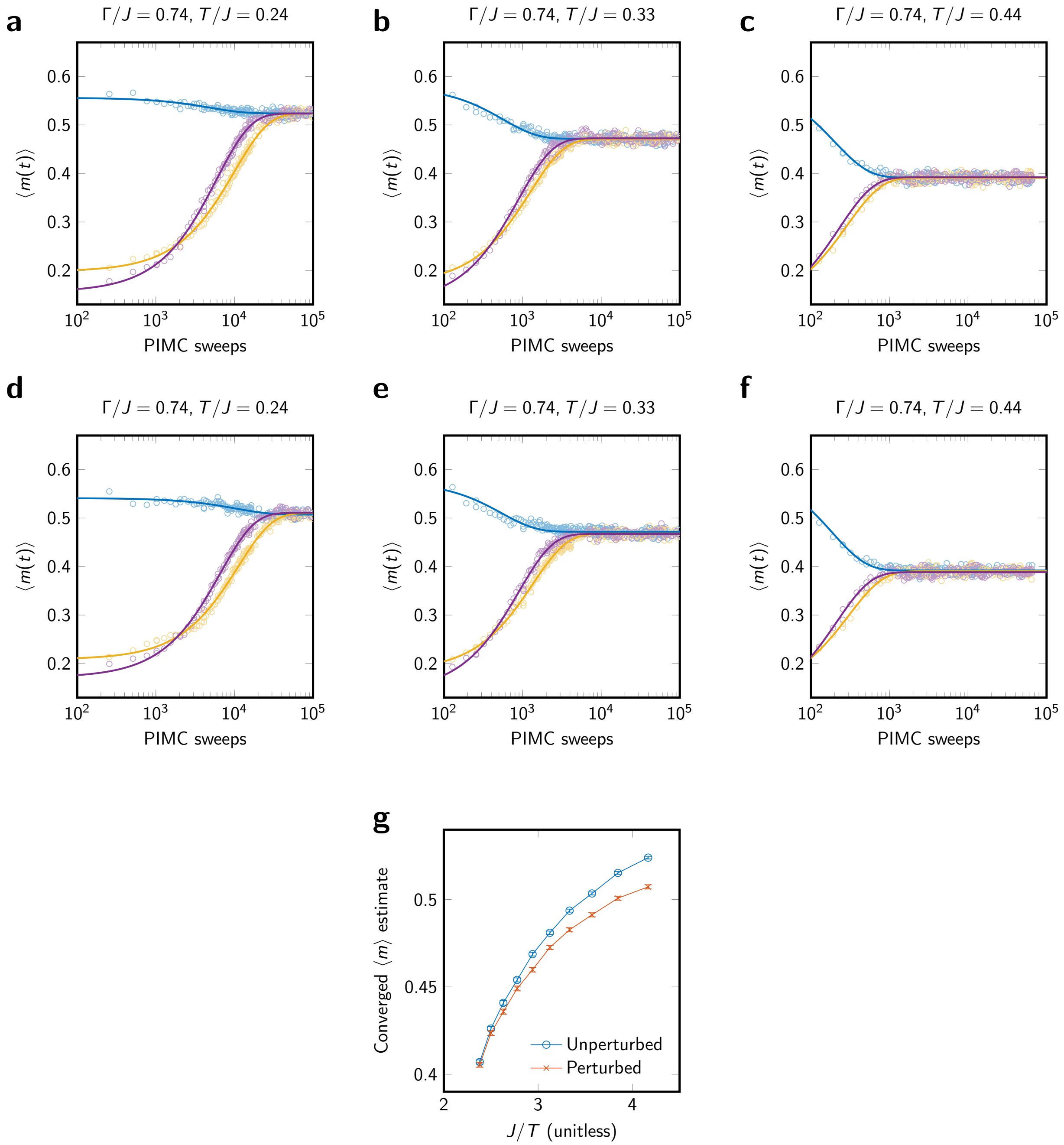

Supplementary FIG. 16. **Effect of disorder in the Ising Hamiltonian.** We show projected PIMC measurements as they converge in the original Hamiltonian (**a**–**c**) and with static disorder added to the Hamiltonians (independent Gaussian errors on the terms with $\sigma = 0.02$) added to the Ising Hamiltonian (**d**–**f**). Symbols are average measurements and lines are exponential fits. Equilibrium estimates of $\langle m \rangle$ (**g**) show that disorder suppresses $\langle m \rangle$, with a temperature-dependent effect that is largest at low temperatures. This is consistent with QA data and demonstrates a disordering effect that does not accelerate convergence. All error bars are 95% confidence interval on the mean.

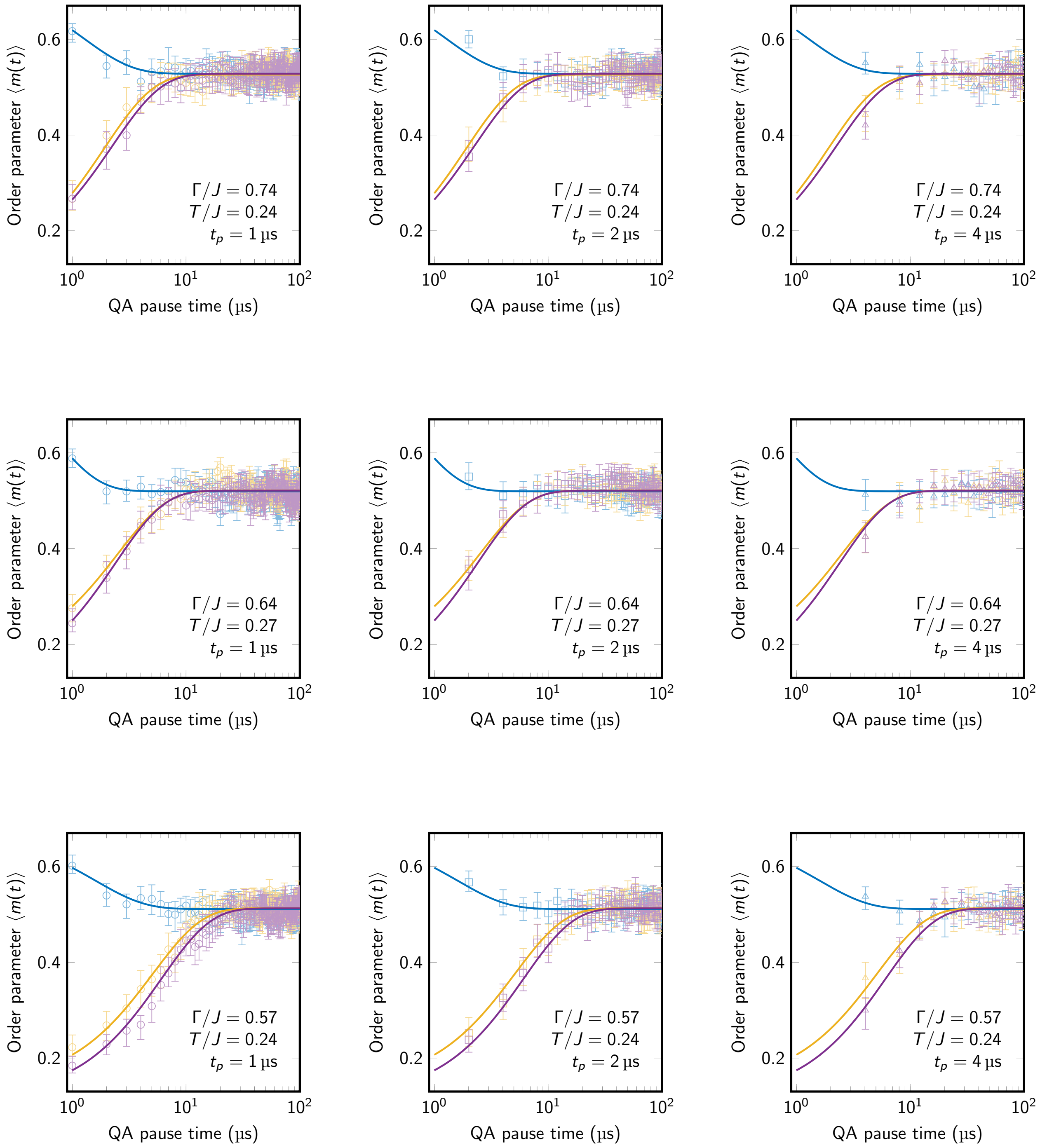

Supplementary FIG. 17. **QA convergence for varying parameters and pause times.** We show QA measurements as in Fig. 2b with different values of pause time t_p separated. Each row is a different value of $(\Gamma/J, T/J)$. For each row, the same exponential fit is shown. All error bars are 95% confidence interval on the mean.

PIMC does not depend on the quench.

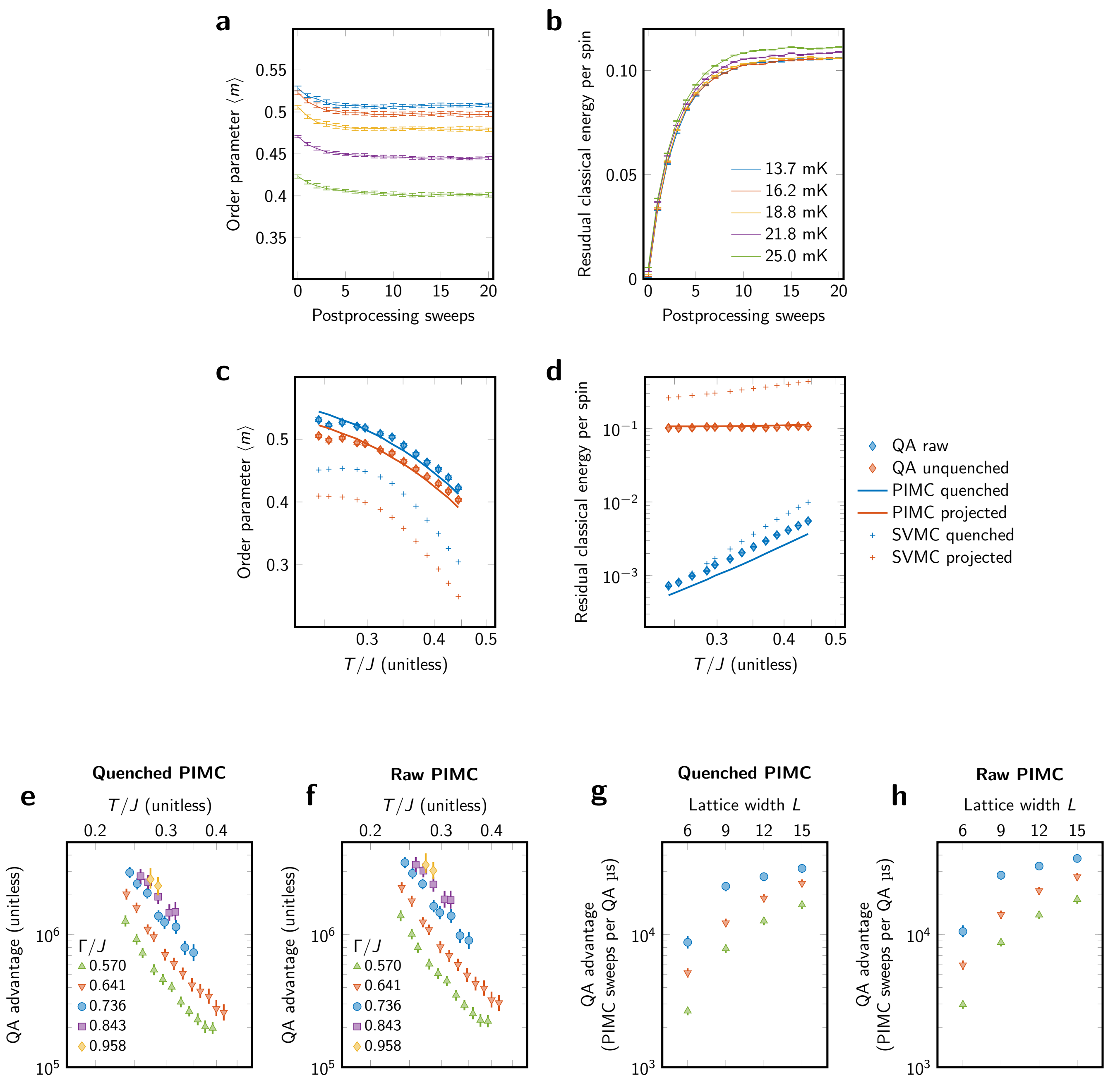

Supplementary FIG. 18. **Quenching and unquenching samples.** **a**–**b**, To mimic a reversal of the QA readout quench, we apply a small number of PIMC postprocessing sweeps to QA output samples. **a**, The effect on $\langle m \rangle$ is modest, and similar in magnitude to the effect of a classical quench on PIMC output samples. **b**, The effect on classical residual energy is significant and is mostly complete after 10 sweeps. **c**–**d**, Quenching PIMC output and unquenching QA output (with 10 sweeps) allows us to compare quenched and unquenched statistics for a range of temperatures with $\Gamma/J = 0.736$. We compare QA results to both PIMC and SVMC (a semiclassical rotor model). **c**, In both QA and PIMC, quenched estimates of $\langle m \rangle$ are roughly 0.02 higher than unquenched estimates. QA and PIMC agree across the range of parameters; SVMC deviates significantly. **d**, Residual classical energy differs dramatically between quenched and unquenched samples in all models, indicating its sensitivity to local processes and therefore the short timescale of the physical QA quench. **e**–**h**, We use quenched and raw PIMC samples to reproduce Fig. 4c (**e**–**f**) and Fig. 4d (**g**–**h**); our main conclusions regarding QA and PIMC relaxation rates are not affected by whether or not we quench PIMC samples. All error bars are 95% confidence interval on the mean.

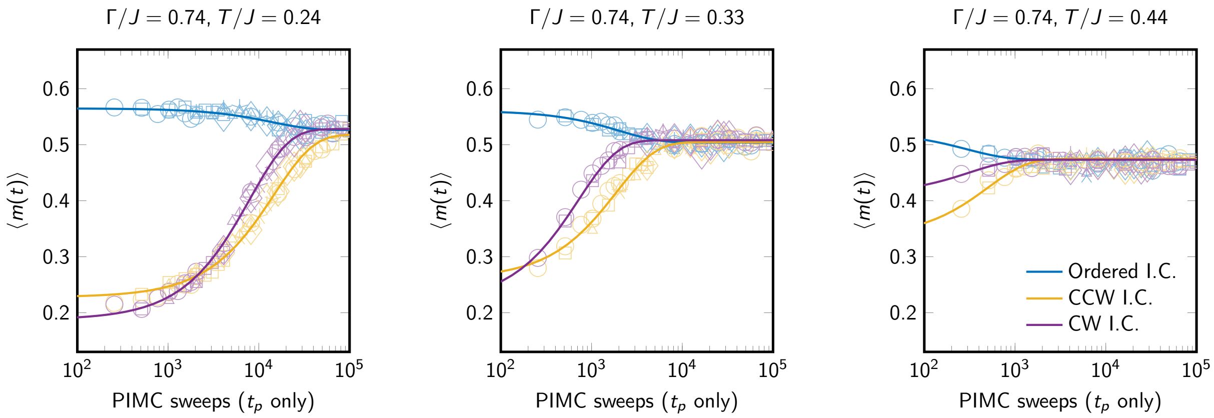

Supplementary FIG. 19. **Modeling QA quench in PIMC.** We assign t_q to be 1000 PIMC sweeps, estimating an equivalent rate from QA data. We then vary t_p in PIMC sweeps (see Supplementary Fig. 7). Different markers indicate different pause lengths t_p; we perform eight steps of each length.

a. *Effect of quench on relaxation timescales*

The PIMC quench we have used up to this point is a simple one-way postprocessing that does not influence the dynamics of PIMC itself. Figs. 7 and 17 show that QA convergence timescales are not strongly affected by the QA quench protocol, so it is appropriate to compare QA dynamics against the dynamics of fixed-Hamiltonian PIMC, only using the quench to roughly quantify the effect of local relaxation. However, the effect of quenching in PIMC is still interesting. To investigate another model of PIMC quench that can potentially affect dynamics, we assume an equivalent PIMC-to-QA rate of 1000 sweeps per QA microsecond. This is a reasonable approximate value (Fig. 4c), and allows us to model the full QA protocol of reverse annealing and quenching. Results are shown in Supplementary Fig. 19. Adopting this more complex PIMC quench does not provide a speedup; the difference in convergence time between $T/J = 0.24$ and $T/J = 0.33$ remains just under an order of magnitude (cf. Fig. 4a). In addition, $\langle m \rangle$ is overestimated far more at $T/J = 0.44$ than in QA.

We also explore an alternative PIMC quench method that differs from the QA protocol. Instead of quenching both thermal and quantum fluctuations as in QA (and Supplementary Fig. 19), we quench only Γ, linearly over a few PIMC sweeps. We perform this quench with free parameters t_p and t_q rather than with parameters motivated by the QA protocol. Supplementary Fig. 20 shows data in which each sample is derived from the previous by Γ-only reverse anneal and quench of duration $t_q = 10$ sweeps, with a pause of $t_p \in \{10, 100, 1000\}$ sweeps. Note that this is more frequent than QA quench in our experiments. The data shows that fast quenches in PIMC can lead to a highly non-equilibrium dynamics and can provide speedup. Data is qualitatively similar for $t_q = 0$ sweeps, where PIMC is periodically projected to the classical space. Pause length t_p provides a tradeoff between escape dynamics and simulation accuracy; for $t_p = 100$ the effect on escape dynamics is large compared to the effect on $\langle m \rangle$. We hypothesize that the dynamical effect is related to the fact that when $\Gamma = 0$ and $T > 0$, the effective Hamiltonian for four-qubit PIMC updates is a classical triangular Ising antiferromagnet, which has no order-by-disorder effect compelling neighboring pseudospins to align exactly.

8. Local signatures of entanglement

Entanglement is a property restricted to quantum systems, and has been demonstrated in a quantum annealing processor similar to the one used in this study[24]. At finite temperatures the equilibrium distribution of a quantum system is described by a mixed state, and this makes unambiguous establishment of entanglement difficult. Here we examine two entanglement witnesses: bipartite concurrence[43] and the Peres-Horodecki criterion[44,45] (positive partial transpose), both of which demonstrate interesting forms of entanglement that place limitations on the ability of quasi-classical models to simulate the system. Significant entanglement is present at the temperature and transverse field ranges examined; these simple measures indicate increased entanglement with decreasing temperature over the experimental range. This provides a plausible explanation—increasing entanglement—for the increasing computational advantage seen in the quantum hardware as temperature decreases.

Certain forms of bipartite entanglement can also be measured in large systems by a quantum Monte Carlo method.

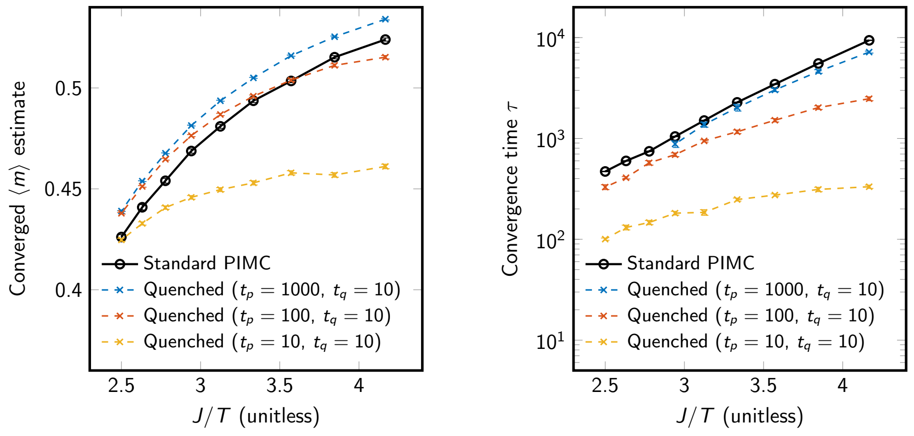

Supplementary FIG. 20. **Effect of alternative quench methods in PIMC.** Supplementary Fig. 19 modeled QA quench in PIMC using approximately corresponding, physically motivated parameters. Here we explore an alternative approach, quenching only Γ/J instead of both T/J and Γ/J, and we do so more frequently and quickly than corresponding QA parameters. At $\Gamma = 0$ and $T > 0$ the system, like the triangular Ising antiferromagnet, has no long range order. Consequently at low temperatures there is a temperature-dependent effect on order (left) and acceleration of escape (right). Quench frequency provides a tradeoff between speed of escape and simulation accuracy. Error bars are from exponential fitting.

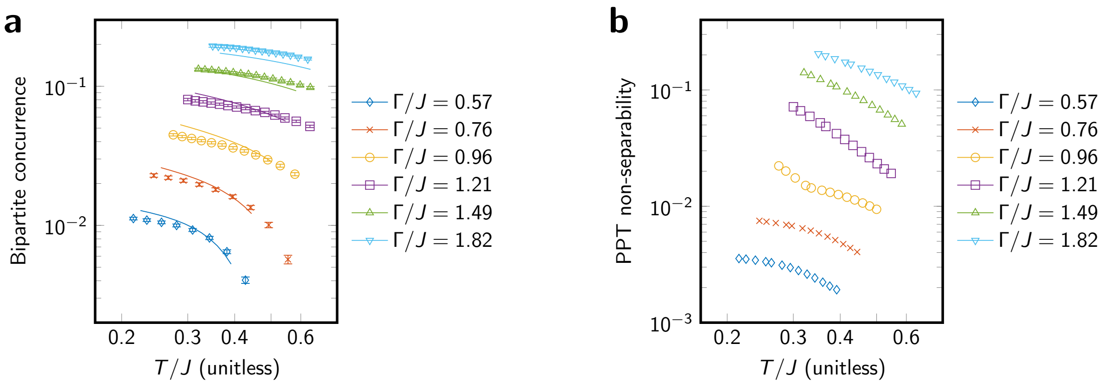

Supplementary FIG. 21. **Entanglement witnesses. a**, We consider bipartite concurrence as a witness of entanglement between two central spins in a four-qubit chain. Lines indicate exact diagonalization results on the 12-qubit gadget (Supplementary Fig. 10a), and marks indicate estimates from quantum Monte Carlo on central chains in a cylindrical lattice with $L = 6$. Concurrence increases with $1/T$ and with Γ in the experimental parameter range. **b**, By considering the smallest eigenvalue (most negative) associated to the 12-qubit gadget instance under partial transpose of one 4-qubit block, we can establish a witness for entanglement between chains. The absolute value of the negative eigenvalue is plotted; as expected it decreases with increasing temperature, and peaks at intermediate transverse field, indicating the failure of factorized approximations (mapping four-qubit chains to logical spins) throughout the experimental range. This measure is limited to the 12-qubit gadget due to the computational complexity of the witness on larger systems. All error bars are 95% confidence interval on the mean.

Many terms in the density matrix can be estimated using standard PIMC; measurement of certain off-diagonal terms—as a ratio of partition functions—requires modification of the imaginary time boundary conditions. By doing so we can measure traced density matrices in large systems and establish bipartite concurrence in our larger lattices. Bipartite entanglement results for neighboring central spins on four-qubit chains are shown in Supplementary Fig. 21a. We find strong concurrence at the level of pairs across the experimental parameter range. We show this both in the 12 qubit gadget system, and in the $L = 6$ lattice under investigation. This result contrasts with the perturbative argument for the nature of entanglement, which is based only on the presence of GHZ states. The presence of entanglement between two qubits in a chain, after tracing out the other two, indicates rich quantum correlations beyond the perturbative argument. Thus we establish not only concurrence, but that its form is notably distinct from that predicted in the perturbative regime across the parameters studied.

We also applied this entanglement measure to qubits beyond chains at various distances, but did not find an unambiguous signal for quantum correlations. Tracing out of variables is necessary for a practical method at scale

based on this entanglement witness, but in the process evidence of entanglement can be destroyed. However, we can use alternative witnesses over small systems. The absence of entanglement allows for factorization of the distribution with respect to chains. To test this we can use the Peres-Horodecki criterion at the level of a chain; a partial transpose with respect to the chain will yield no negative eigenvalues if there exists a separable (quasi-classical) approximation. As shown in Supplementary Fig. 21b, this criterion indicates the presence of entanglement beyond the chain level throughout the experimental range, with the evidence becoming weaker at larger temperature and smaller Γ as expected.

It should be noted that the bipartite concurrence and Peres-Horodecki witnesses chosen to demonstrate entanglement here are sufficient, but not necessary, for the demonstration of entanglement and we have not been able to link them to a specific accelerating dynamical mechanism. Developing and applying witnesses better tailored to the lattice structure and phases, and exploiting only QA-accessible information, is future work.

9. Additional observables

Here we provide data on additional observables. Supplementary Fig. 22 shows convergence and equilibrium values of winding number. Both PIMC and QA exhibit asymmetry between counterclockwise and clockwise winding that varies as a function of T/J and Γ/J. This asymmetry is a subtle detail of equilibrium statistics in the square-octagonal lattice that is absent in the related triangular antiferromagnet, where there exists an additional flip symmetry of the cylinder. It is important for two reasons. First, it indicates that the quantum simulation is accurate enough to exhibit highly nontrivial physics, thereby demonstrating the utility of quantum simulation. Second, it immediately rules out several simplified models of the lattice such as the triangular AFM, six-state clock model, and any other model from which this asymmetry is absent.

Supplementary Fig. 23 shows two-point pseudospin correlations along the periodic axis of the cylinder, taken far from the open boundaries as in previous work[2]. The decay form of these correlations are important witnesses of critical phenomena[2,5].

Supplementary Fig. 24 reproduces the data in Fig. 3b–c for smaller instances, showing accuracy in estimating $\langle m \rangle$ for every system size studied, usually to within 0.01 of converged PIMC estimates.

10. Source code

Source code is available for reproduction of our work under an Apache license. This takes the form of a C++ class. The latest version of the source code is available at `https://github.com/dwavesystems/dwave-pimc`. The code is written for generic sparse transverse field Ising models with fixed transverse field

$$\hat{H} = \sum_{i,j} J_{i,j} \sigma_i^z \sigma_j^z + \sum_i \left[h_i \sigma_i^z - \Gamma \sigma_i^x \right] . \tag{7}$$

Code generates projected samples at any temperature. The number of slices is set to 2^{16} as a numerically convenient approximation to infinity appropriate for practical parameterizations.

A single qubit method is provided for generic models. For models where blocks of size X form ferromagnetic chains with uniform coupling, as in the square-octagonal case ($X = 4$), a *chain* update is also provided. The chain update significantly accelerates equilibration for this model, as discussed in the paper. Square-octagonal and triangular lattices—along with their CW/CCW and ordered initial conditions—can be prepared, and the three-sublattice partitioning of variables required for inference of the order parameter m is provided.

Results as measured in sweeps can be reproduced by this code, Figs. 11(f) and 14 for example. For simplicity we have provided a reduced-feature code, with certain optimizations removed. These have a small impact on performance; the wall-clock times that define Figs. 2 and 11(f) remain reasonably well approximated. The timescales assocated to PIMC applied to the triangular lattice in Section 5 a, were slower by a factor of approximately 5.

* aking@dwavesys.com
† These authors contributed equally.

[1] P. I. Bunyk, E. M. Hoskinson, M. W. Johnson, E. Tolkacheva, F. Altomare, A. J. Berkley, R. Harris, J. P. Hilton, T. Lanting, A. J. Przybysz, and J. Whittaker, IEEE Transactions on Applied Superconductivity **24**, 1 (2014).

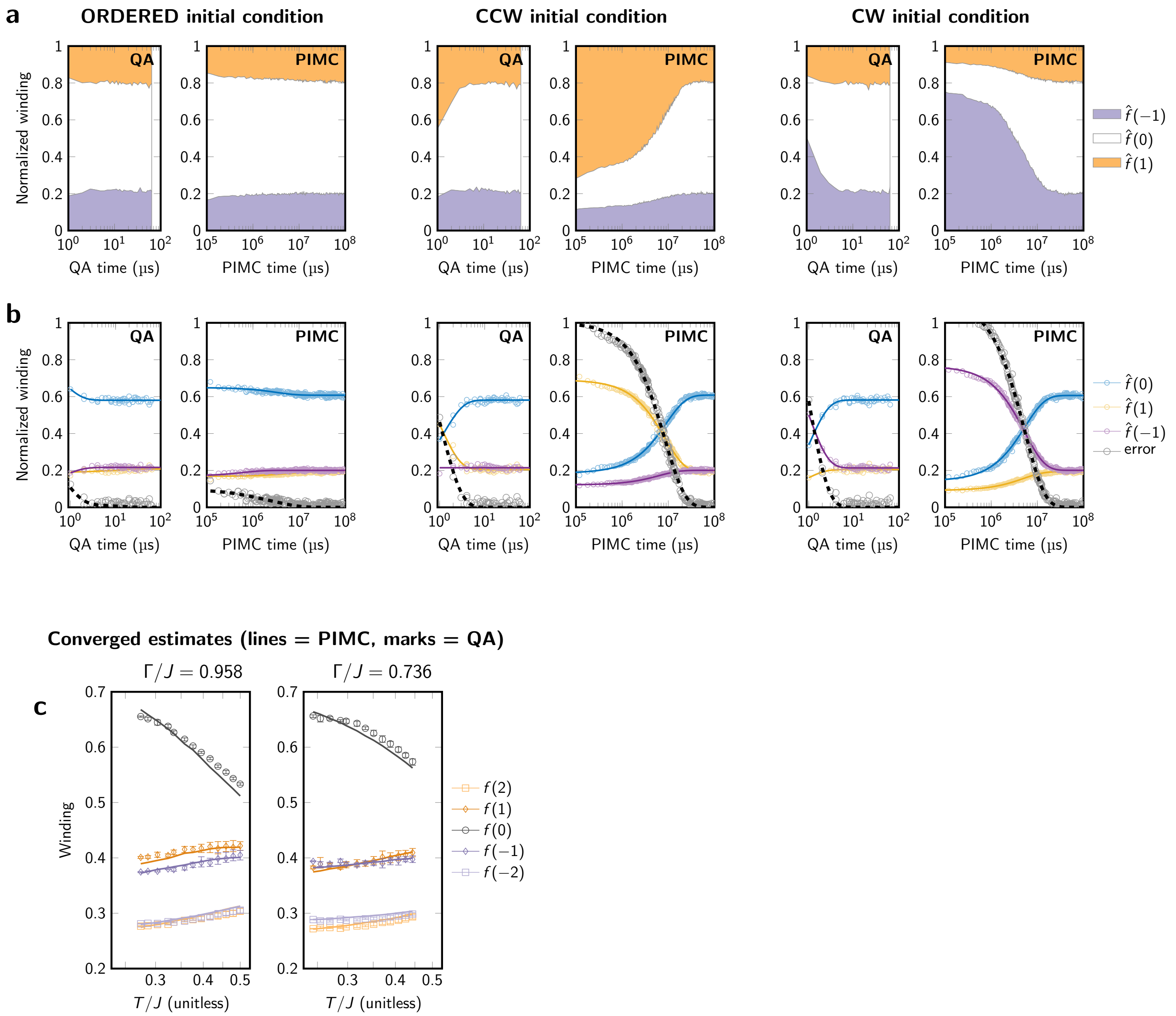

Supplementary FIG. 22. **Convergence of winding number in QA and PIMC.** Analogous to the data presented in Fig. 2b, we can observe convergence of winding number $f(w)$ from various initial conditions, in QA and PIMC (quenched). Data shown is for $f(0)$, $f(1)$, and $f(-1)$, normalized by their sum to give $\hat{f}(w) = f(w)/(f(0) + f(1) + f(-1))$. **a**, Area plots of the three normalized winding numbers $\hat{f}(w)$ shows convergence to a stable distribution for each simulation regardless of initial condition. **b**, Normalized winding numbers $\hat{f}(w)$ are plotted separately, along with exponential fit functions. The sum of these fits' deviations from their equilibrium values gives a measure of error on the distribution, which converges to zero. As in Fig. 2b, data shown correspond to $\Gamma/J = 0.736$, $T/J = 0.244$. **c**, Equilibrium estimates of non-normalized winding numbers $f(w)$ for QA (markers) and PIMC (lines) are shown at two values of Γ/J corresponding to $s = 0.36$ and $s = 0.38$. Beyond the broad quantitative agreement, QA accurately captures subtle equilibrium properties of the square-octagonal lattice, in particular the asymmetry between negative and positive winding and its dependence on Γ and T. Error bars are 95% bootstrap confidence intervals.

[2] A. D. King, J. Carrasquilla, J. Raymond, I. Ozfidan, E. Andriyash, A. J. Berkley, M. Reis, T. Lanting, R. Harris, F. Altomare, K. Boothby, P. I. Bunyk, C. Enderud, A. Fréchette, E. M. Hoskinson, N. Ladizinsky, T. Oh, G. Poulin-Lamarre, C. Rich, Y. Sato, A. Y. Smirnov, L. J. Swenson, M. H. Volkmann, J. Whittaker, J. Yao, E. Ladizinsky, W. Mark, J. P. Hilton, and M. H. Amin, Nature **560**, 456 (2018).

[3] R. Moessner, S. L. Sondhi, and P. Chandra, Physical Review Letters **84**, 4457 (2000).

[4] R. Moessner and S. L. Sondhi, Physical Review B **63**, 1 (2001).

[5] S. V. Isakov and R. Moessner, Physical Review B **68**, 104409 (2003).

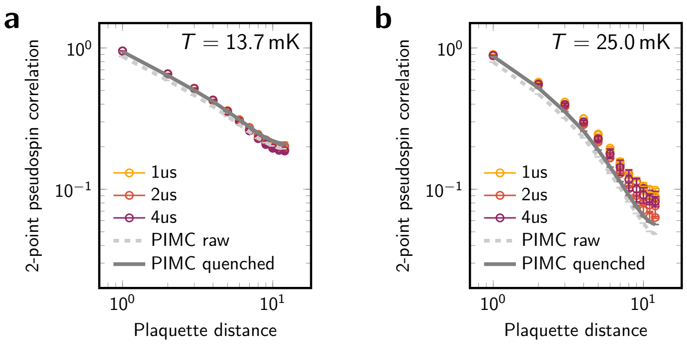

Supplementary FIG. 23. **Two-point correlation functions.** We measure two-point pseudospin correlations along the periodic axis of the cylinder for the 1440-spin lattice and find good agreement between QA and PIMC. Data shown are for $\Gamma/J = 0.736$ as in Fig. 2c–d. Long-range correlations are overestimated for high temperature, consistent with the hypothesis of a limited global ordering in the lattice during the QA readout quench. All error bars are 95% confidence interval on the mean.

[6] K. Damle, Physical Review Letters **115**, 127204 (2015).
[7] Y. Jiang and T. Emig, Journal of Physics: Condensed Matter **19**, 145234 (2007).
[8] S. E. Korshunov, Physical Review B **86**, 014429 (2012).
[9] D. Blankschtein, M. Ma, A. N. Berker, G. S. Grest, and C. M. Soukoulis, Physical Review B **29**, 5250 (1984).
[10] J.-j. Kim, Y. Yamada, and O. Nagai, Physical Review B **41**, 4760 (1990).
[11] R. A. Jalabert and S. Sachdev, Physical Review B **44**, 686 (1991).
[12] G. H. Wannier, Phys. Rev. **79**, 357 (1950).
[13] J. V. José, L. P. Kadanoff, S. Kirkpatrick, and D. R. Nelson, Physical Review B **16**, 1217 (1977).
[14] M. S. S. Challa and D. P. Landau, Physical Review B **33**, 437 (1986).
[15] V. L. Berezinskii, Sov. Phys. JETP **34**, 610 (1972).
[16] J. M. Kosterlitz and D. J. Thouless, Journal of Physics C: Solid State Physics **6**, 1181 (1973).
[17] H. Li, Y. D. Liao, B.-B. Chen, X.-T. Zeng, X.-L. Sheng, Y. Qi, Z. Y. Meng, and W. Li, Nature Communications **11**, 1111 (2020).
[18] R. Harris, Y. Sato, A. J. Berkley, M. Reis, F. Altomare, M. H. Amin, K. Boothby, P. I. Bunyk, C. Deng, C. Enderud, S. Huang, E. M. Hoskinson, M. W. Johnson, E. Ladizinsky, N. Ladizinsky, T. Lanting, R. Li, T. Medina, R. Molavi, R. Neufeld, T. Oh, I. Pavlov, I. Perminov, C. Rich, A. Smirnov, L. Swenson, N. Tsai, M. Volkmann, J. Whittaker, and J. Yao, Science **165**, 162 (2018).
[19] T. Kadowaki and H. Nishimori, Physical Review E **58**, 5355 (1998).
[20] E. Farhi, J. Goldstone, S. Gutmann, J. Lapan, A. Lundgren, and D. Preda, Science **292**, 472 (2001).
[21] T. Albash and D. A. Lidar, Reviews of Modern Physics **90**, 015002 (2018).
[22] R. Harris, J. Johansson, A. J. Berkley, M. W. Johnson, T. Lanting, S. Han, P. Bunyk, E. Ladizinsky, T. Oh, I. Perminov, E. Tolkacheva, S. Uchaikin, E. M. Chapple, C. Enderud, C. Rich, M. Thom, J. Wang, B. Wilson, and G. Rose, Physical Review B **81**, 1 (2010).
[23] A. J. Berkley, A. J. Przybysz, T. Lanting, R. Harris, N. Dickson, F. Altomare, M. H. Amin, P. Bunyk, C. Enderud, E. Hoskinson, M. W. Johnson, E. Ladizinsky, R. Neufeld, C. Rich, A. Y. Smirnov, E. Tolkacheva, S. Uchaikin, and A. B. Wilson, Physical Review B **87**, 1 (2013).
[24] T. Lanting, A. J. Przybysz, A. Y. Smirnov, F. M. Spedalieri, M. H. Amin, A. J. Berkley, R. Harris, F. Altomare, S. Boixo, P. I. Bunyk, N. G. Dickson, C. Enderud, J. P. Hilton, E. M. Hoskinson, M. W. Johnson, E. Ladizinsky, N. Ladizinsky, R. Neufeld, T. Oh, I. Perminov, C. Rich, M. Thom, E. Tolkacheva, S. Uchaikin, A. B. Wilson, and G. Rose, Physical Review X **4**, 021041 (2014).
[25] M. W. Johnson, M. H. Amin, S. Gildert, T. Lanting, F. Hamze, N. G. Dickson, R. Harris, A. J. Berkley, J. Johansson, P. I. Bunyk, E. M. Chapple, C. Enderud, J. P. Hilton, K. Karimi, E. Ladizinsky, N. Ladizinsky, T. Oh, I. Perminov, C. Rich, M. C. Thom, E. Tolkacheva, C. J. S. Truncik, S. Uchaikin, J. Wang, B. Wilson, and G. Rose, Nature **473**, 194 (2011).
[26] M. Suzuki, Progress of Theoretical Physics **56**, 1454 (1976).
[27] H. G. Evertz, Advances in Physics **52**, 1 (2003).
[28] H. Rieger and N. Kawashima, The European Physical Journal B **9**, 233 (1999).
[29] A. W. Sandvik, Physical Review E **68**, 056701 (2003).
[30] S. V. Isakov, G. Mazzola, V. N. Smelyanskiy, Z. Jiang, S. Boixo, H. Neven, and M. Troyer, Physical Review Letters **117**, 180402 (2016).
[31] K. Y. Camsari, S. Chowdhury, and S. Datta, Physical Review Applied **12**, 034061 (2019), arXiv:1810.07144.
[32] S. R. White, Physical Review Letters **69**, 2863 (1992).
[33] A. E. Feiguin and S. R. White, Physical Review B - Condensed Matter and Materials Physics **72**, 2 (2005).
[34] B. B. Chen, L. Chen, Z. Chen, W. Li, and A. Weichselbaum, Physical Review X **8**, 31082 (2018).
[35] P. J. Reynolds, J. Tobochnik, and H. Gould, Computers in Physics **4**, 662 (1990).

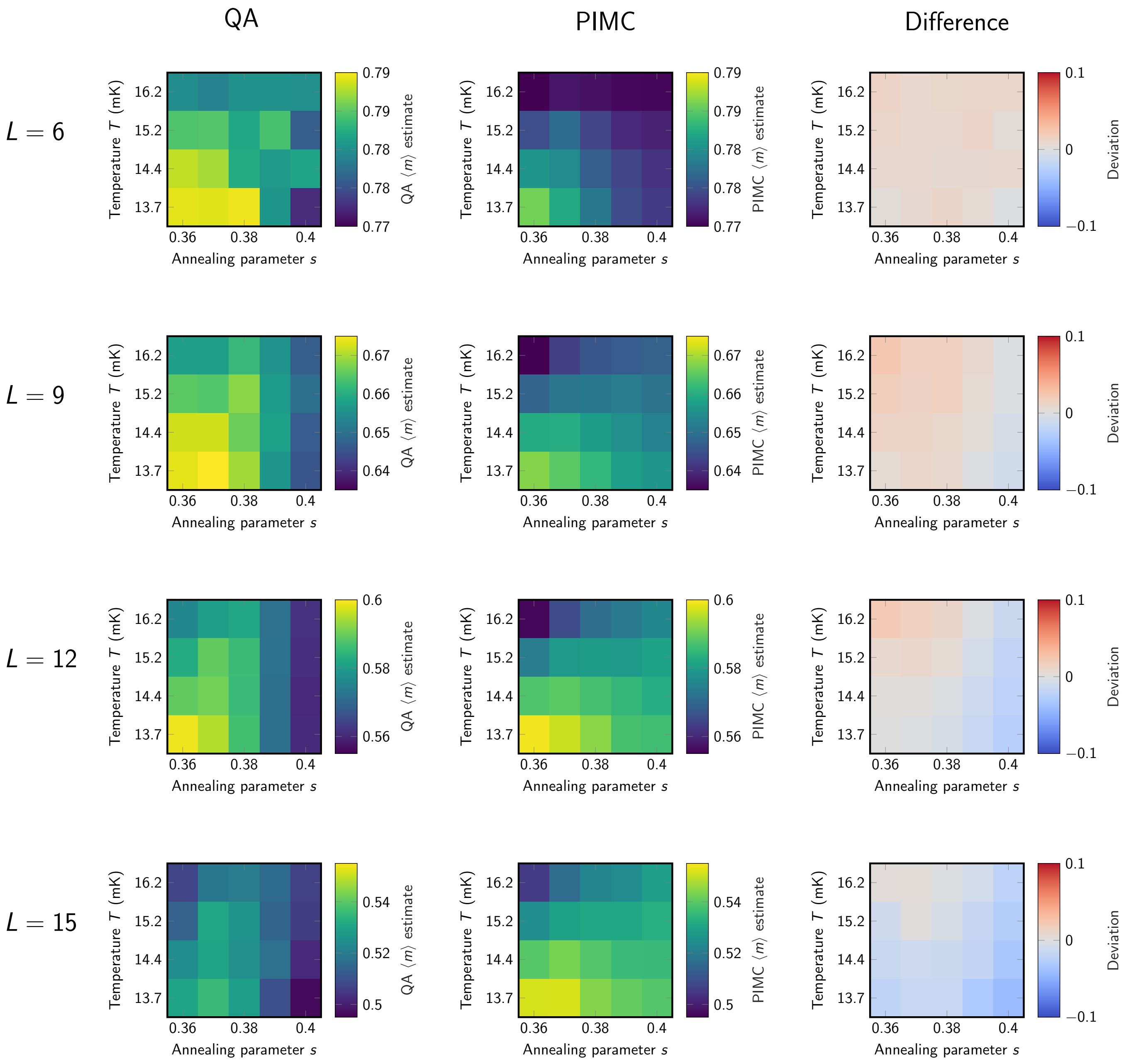

Supplementary FIG. 24. **QA and PIMC equilibrium estimates of order parameter.** Analogous to Fig. 3b and c, we show data for all system sizes studied for the four coldest temperatures and slowest-converging annealing parameters studied. These estimates are derived from an average of ordered and random initial conditions.

[36] G. Carleo and M. Troyer, Science **355**, 602 (2017).
[37] M. Hibat-Allah, M. Ganahl, L. E. Hayward, R. G. Melko, and J. Carrasquilla, Physical Review Research **2**, 023358 (2020).
[38] R. G. Melko, in *Strongly Correlated Systems*, edited by A. Avella and F. Mancini (2013) pp. 185–206.
[39] S. Biswas, G. Rakala, and K. Damle, Physical Review B **93**, 1 (2016), arXiv:1512.00931.
[40] I. Herbut, *A Modern Approach to Critical Phenomena* (Cambridge University Press, Cambridge, 2007).
[41] S. W. Shin, G. Smith, J. A. Smolin, and U. Vazirani, "How "Quantum" is the D-Wave Machine?" (2014), arXiv:1401.7087.
[42] T. Albash, I. Hen, F. M. Spedalieri, and D. A. Lidar, Physical Review A **92**, 1 (2015), arXiv:1506.03539.
[43] S. Hill and W. K. Wootters, Physical Review Letters **78**, 5022 (1997).
[44] A. Peres, Physical Review Letters **77**, 1413 (1996).
[45] M. Horodecki, P. Horodecki, and R. Horodecki, Physics Letters A **223**, 1 (1996).
